# Intercontinental trends in functional and phylogenetic structure of stream fish assemblages

**DOI:** 10.1002/ece3.5823

**Published:** 2019-11-19

**Authors:** Luke M. Bower, Kirk O. Winemiller

**Affiliations:** ^1^ Department of Wildlife and Fisheries Sciences Texas A&M University College Station TX USA

**Keywords:** community assembly, environmental filtering, fish, functional diversity, phylogenetic diversity

## Abstract

Understanding of community assembly has been improved by phylogenetic and trait‐based approaches, yet there is little consensus regarding the relative importance of alternative mechanisms and few studies have been done at large geographic and phylogenetic scales. Here, we use phylogenetic and trait dispersion approaches to determine the relative contribution of limiting similarity and environmental filtering to community assembly of stream fishes at an intercontinental scale. We sampled stream fishes from five zoogeographic regions. Analysis of traits associated with habitat use, feeding, or both resulted in more occurrences of trait underdispersion than overdispersion regardless of spatial scale or species pool. Our results suggest that environmental filtering and, to a lesser extent, species interactions were important mechanisms of community assembly for fishes inhabiting small, low‐gradient streams in all five regions. However, a large proportion of the trait dispersion values were no different from random. This suggests that stochastic factors or opposing assembly mechanisms also influenced stream fish assemblages and their trait dispersion patterns. Local assemblages tended to have lower functional diversity in microhabitats with high water velocity, shallow water depth, and homogeneous substrates lacking structural complexity, lending support for the stress‐dominance hypothesis. A high prevalence of functional underdispersion coupled with phylogenetic underdispersion could reflect phylogenetic niche conservatism and/or stabilizing selection. These findings imply that environmental filtering of stream fish assemblages is not only deterministic, but also influences assemblage structure in a fairly consistent manner worldwide.

## Graphical abstract

Phylogenetic and trait dispersion approaches were used to determine the relative contribution of limiting similarity and environmental filtering to community assembly of stream fishes at an intercontinental scale. Our results suggest that environmental filtering and, to a lesser extent, species interactions were important mechanisms of community assembly for fishes inhabiting small, low‐gradient streams in all five regions. Local assemblages tended to have lower functional diversity in microhabitats with high stress, such as high‐water velocity, shallow water depth, and homogeneous substrates lacking structural complexity, lending support for the stress‐dominance hypothesis.

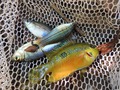

## 1 INTRODUCTION

Ecologists have long sought to understand the mechanisms that account for local community assembly, species coexistence, and functional diversity. Despite the growing need to predict community responses to environmental change, mechanisms underlying community assembly remain poorly understood (Mouillot, Dumay, & Tomasini, 2007; Pavoine & Bonsall, 2011; Weiher et al., 2011). Two assembly processes, environmental filtering and limiting similarity, are generally thought to play important roles in structuring communities (Chase & Myers, 2011; MacArthur & Levins, 1967; Perronne, Munoz, Borgy, Reboud, & Gaba, 2017; Weiher & Keddy, 1995). However, evidence supporting these mechanisms has been inconsistent among studies involving various taxa, spatiotemporal scales, and methodologies. Many studies have inferred environmental filtering, a process whereby local environmental conditions prevent the successful establishment of certain species in a particular habitat (e.g., Córdova‐Tapia, Hernández‐Marroquín, & Zambrano, 2018; Dimitriadis, Evagelopoulos, & Koutsoubas, 2012; Mouchet, Burns, Garcia, Vieira, & Mouillot, 2013; Mouillot et al., 2007; Troia & Gido, 2015; Weiher et al., 2011). Others support limiting similarity, the avoidance of competitive exclusion within a given habitat through niche partitioning, as the dominate process structuring assemblages (e.g., Ingram & Shurin, 2009; Montaña, Winemiller, & Sutton, 2014; Weiher & Keddy, 1995). To improve our ability to predict biodiversity responses to environmental change, research is needed to reveal how environmental variation influences mechanisms of community assembly and resultant structures.

**Figure 1:**
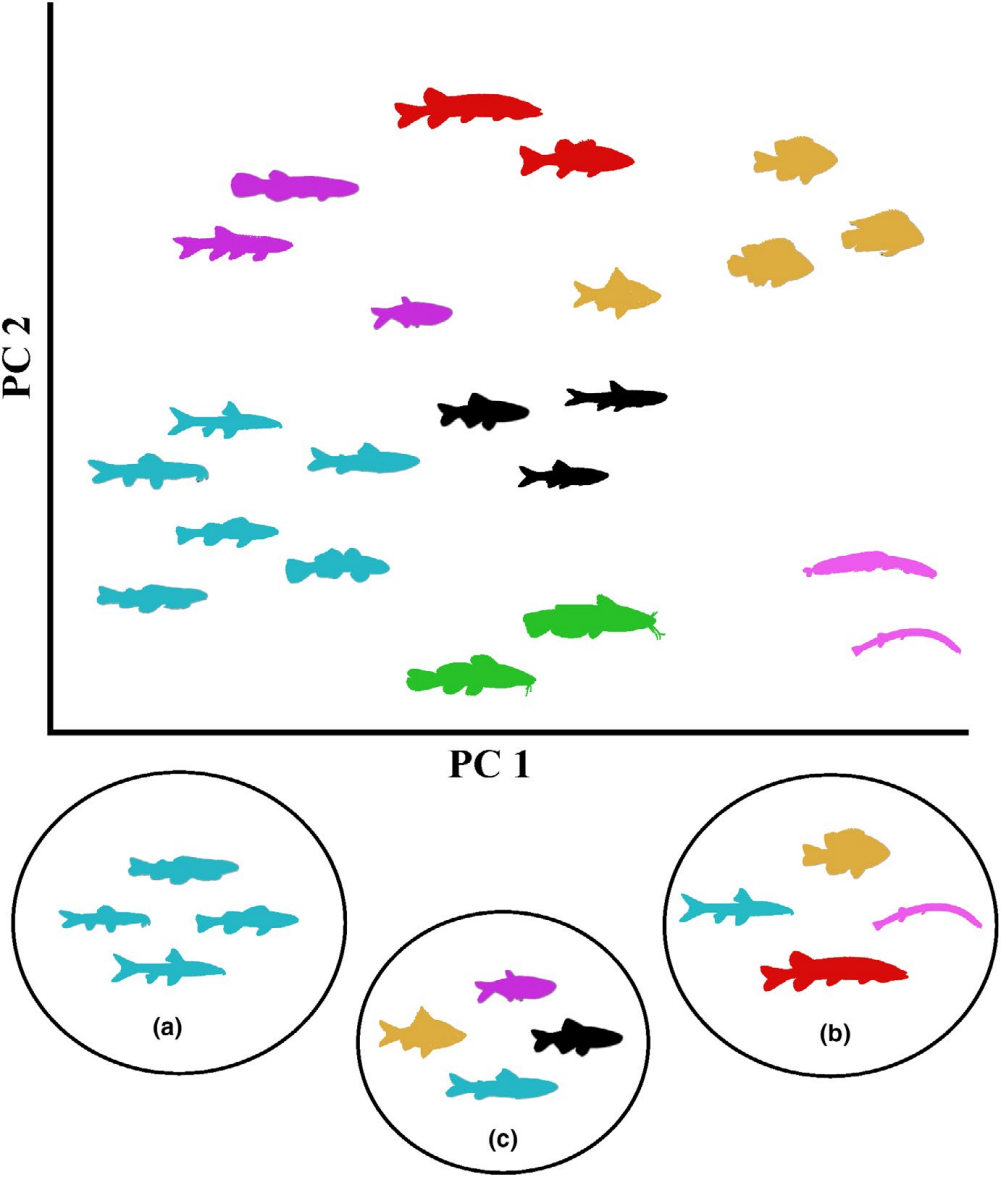
Two PC axes depicting a theoretical morphospace of region species pool. Colors represent different fish niches or ecomorphological groups. Circles represent the local species pool within microhabitat habitats, illustrating (a) environmental filtering resulting in underdispersion of traits; (b) limiting similarity resulting in overdispersion of traits; (c) limiting similarity acting on a local species pool after environmental filtering has occurred, resulting in trait overdispersion of species with similar habitat requirements

Trait‐based and phylogenetic methods have been increasingly used to disentangle the mechanisms that influence community assembly (Mouillot et al., 2007; Swenson, 2013; Violle, Reich, Pacala, Enquist, & Kattge, 2014). Species assemblages influenced by environmental filtering are expected to have trait distributions that are narrower, or underdispersed, than expected at random, because only those species with traits suited for the environment can establish and persist (Figure 1; Cornwell & Ackerly, 2009; Weiher & Keddy, 1995). Alternatively, competition and limiting similarity should result in an assemblage trait distribution that is overdispersed compared to random (Figure 1; Brown & Wilson, 1956; MacArthur & Levins, 1967; Weiher & Keddy, 1995). These interpretations are not always straightforward. In some circumstances, interspecific competition could yield functional trait underdispersion, such as when plants have similar heights due to competition for sunlight (Mayfield & Levine, 2010). When studies combine traits associated with different niche dimensions into a single analysis, independent effects of separate dimensions may be obscured, resulting in erroneous conclusion that neutral mechanisms play the dominant role in community assembly (Kraft, Cornwell, Webb, & Ackerly, 2007; Weiher et al., 2011). For example, Trisos, Petchey, and Tobias (2014) found that datasets representing multiple niche axes had low power for detecting community assembly processes, but single niche axes were better able to detect the signals of environmental filtering and limiting similarity in bird assemblages. By focusing on different niche dimensions, studies should be able to parse out the influences of different community assembly processes. Therefore, interpreting patterns of over‐ and underdispersion is challenging and requires considerable system‐specific knowledge to inform study design, analysis, and inference (Mayfield & Levine, 2010).

Spatial scale also is an important aspect of study design for research on community assembly, because community assembly processes are expected to change along spatial hierarchies (Blanchet, Helmus, Brosse, & Grenouillet, 2014; Levin, 1992; Oberdoff, Guégan, & Hugueny, 1995; Poff, 1997; Smith, Sandel, Kraft, & Carey, 2013). At broad spatial scales (regional to global), abiotic environmental filters should have the greatest influence on community structure, affecting processes such as speciation, dispersal, and extinction. At local scales, community assembly and population persistence are heavily influenced by both abiotic environmental variation and biotic factors such as productivity and species interactions (Algar, Kerr, & Currie, 2011; Brooker et al., 2009; Weiher et al., 2011). Some studies have suggested that analysis at finer spatial resolution shifts the dominant community assembly process from environmental filtering to limiting similarity (Götzenberger et al., 2012; Montaña et al., 2014; Vamosi, Heard, Vamosi, & Webb, 2009; Weiher et al., 2011; Weiher & Keddy, 1995). Community assembly processes also may vary according to levels of environmental stress (Coyle et al., 2014; Ramm et al., 2018; Swenson & Enquist, 2007; Weiher & Keddy, 1995). For example, squamate assemblages from arid regions of Africa displayed characteristics consistent with environmental filtering to a greater degree than those from wet tropical regions (Ramm et al., 2018). Ascertaining how assemblage structure changes along environmental gradients at different spatial scales can reveal how alternative processes influence community assembly.

Despite intense interest in community assembly processes, mechanisms and rules that apply across different systems have not been identified. This lack of fundamental understanding may derive from three possibilities (HilleRisLambers, Adler, Harpole, Levine, & Mayfield, 2012; McGill, Enquist, Weiher, & Westoby, 2006). First, much of the research on functional diversity patterns has been focused on plants and microbes, with relatively few studies on animals (Trisos et al., 2014). Thus, our understanding of community assembly processes has largely been based on organisms with limited mobility. Second, investigations of functional diversity patterns across large spatial scales are rare, especially for vertebrates in aquatic systems (Heino et al., 2013; Troia et al., 2015). For example, functional diversity studies of fish assemblages often focus on only one zoogeographic region, preventing the comparison of phylogenetically distinct assemblages. Studies across large geographic scales are essential for identifying general patterns of ecology (Coyle et al., 2014; Pianka, Vitt, Pelegrin, Fitzgerald, & Winemiller, 2017; Ramm et al., 2018). Third, discrepancies in methods of data collection and analysis complicate comparisons based on meta‐analysis of functional traits and phylogenetic diversity. Simultaneous analysis of phylogenetic and trait patterns is essential to determine the relationships between the two, and for inferences regarding community assembly (Gerhold, Cahill, Winter, Bartish, & Prinzing, 2015; Ramm et al., 2018; Troia & Gido, 2015).

Harsh conditions should increase the influence of environmental filtering on community assembly (Weiher & Keddy, 1995). The stress‐dominance hypothesis predicts that functional trait diversity will be reduced as environmental stress and stabilizing selection increase, whereas interspecific trait variation is expected to be greater in less stressful environments (Coyle et al., 2014; Ramm et al., 2018; Swenson & Enquist, 2007; Weiher & Keddy, 1995). Stream fishes provide an excellent model system to test this hypothesis. Environmental filters structure stream fish assemblages and can act over multiple spatial and temporal scales (Hoeinghaus, Winemiller, & Birnbaum, 2007; Poff, 1997; Poff & Allan, 1995). For stream fishes, high water velocity is a strong environmental stressor that influences their ecology and evolution (Bower & Piller, 2015; Haas, Heins, & Blum, 2015; Lamouroux, Poff, & Angermeier, 2002; Lujan & Conway, 2015; Willis, Winemiller, & Lopez‐Fernandez, 2005) because hydraulic drag associated with fast‐moving water exerts a high energetic cost (Webb, 1988). Substrate characteristics in streams also affect fish ecology in multiple ways (Kovalenko, Thomaz, & Warfe, 2012). Structurally complex substrates can provide refuge from adverse environmental conditions, such as hydraulic drag or predation (Bartholomew, Diaz, & Cicchetti, 2000; Tokeshi & Arakaki, 2012). Streams with unstructured substrates tend to have fish assemblages with lower functional trait diversity (Bower & Winemiller, 2019; Kovalenko et al., 2012).

Here, we investigate the functional and phylogenetic structure of stream fishes along environmental gradients in five zoogeographic regions. Our first objective was to evaluate the similarity of functional and phylogenetic dispersion patterns across regions at microhabitat and stream‐reach scales. We hypothesize a shift from a strong signal of environmental filter with no signal of limiting similarity at the regional spatial scale, to strong signals of both limiting similarity and environmental filtering at the microhabitat scale. We also hypothesize to find more instances of limiting similarity using traits associated with resource acquisition and higher detection of environmental filtering using traits associated with habitat use. Our second objective was to test the relationship between environmental gradients and metrics of functional trait and phylogenetic diversity. We hypothesized that functional diversity metrics would decline with increasing water velocity but increase with water depth and substrate complexity.

## 2 METHODS

### 2.1 Data acquisition and preparation

**Figure 2:**
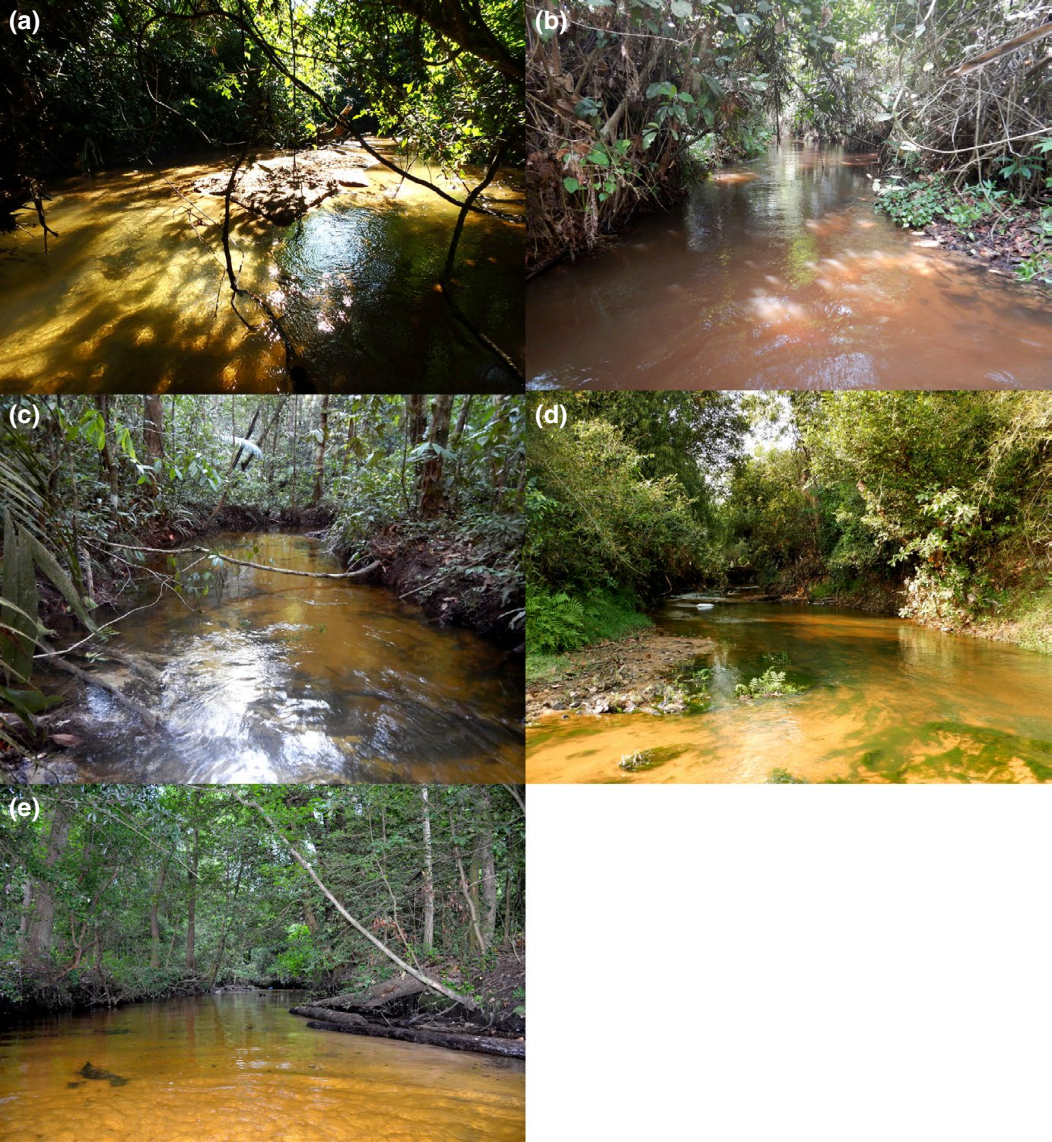
Photographs showing the similarity in streams from each regions: (a) Belize, (b) Benin, (c) Brazil, (d) Cambodia, and (e) USA

Stream fish assemblages were surveyed from five zoogeographic regions on four continents—Belize, Benin, Brazil, Cambodia, and United States of America (New Jersey, South Carolina, and Texas). The inclusion of different zoogeographic regions allows for comparison of distantly related lineages and the testing of general, repeated patterns in community assembly processes. In an attempt to minimize differences in habitat features, only streams with the following characteristics were chosen: (a) low stream order with small channel; (b) low level of disturbance (few anthropogenic impacts); (c) low gradient; (d) within coastal plains or inland floodplains, and (e) geomorphology with meandering channel and sandy substrate (Figure 2). In each region, we sampled five to seven wadeable streams encompassing channel widths classified as small (<3 m), medium (3–8 m), and large (>8 m). Fishes were collected under base‐flow conditions when streams were wadeable and capture was most efficient. To account for spatial‐scale dependency (Smith et al., 2013), a nested sample design was used: microhabitat unit within stream reach within zoogeographic region.

Within each region, stream reaches measuring from 200 to 500 m were sampled in an upstream direction to obtain representative samples of fishes from major types of microhabitat (see Bower & Winemiller, 2019). Microhabitat types were areas of relatively homogeneous depth, current velocity, substrate composition, and in‐channel cover. In each microhabitat where fish were collected, we recorded water velocity, substrate composition, and depth. Microhabitats were sampled only if they fit one of these substrate categorizes: sand (>90% cover), woody structure (>80% cover), aquatic macrophytes (>80% cover), leaf packs (>80% cover), root banks (banks with dense root structures, >90%), and gravel (6‐25 cm diameter, >80% cover). Given the challenge of sampling fish from diverse habitats, various methods were employed, including seining, cast netting, dip‐netting, and backpack electrofishing at each sample site. At each study site, water temperature (°C), dissolved oxygen (DO), pH, specific conductivity (µS/cm), and salinity (ppt) were measured. Specimens were euthanized via anesthetic (MS222) overdose and then preserved in 10% formalin following Texas A&M University animal care protocols IACUC 2014‐0173 and 2017‐0233.

Twenty‐seven morphometric traits that affect food acquisition and locomotion were measured for 5 individuals per species (for rare species, *n* = 1–4; sample sizes appear in Table S1) to the nearest 0.1 mm using calipers (Gatz, 1979; Winemiller, 1991; Table 1). To reduce potential ontogenetic biases, only adult size classes were used for all analyses. Measurements of morphological components were standardized by converting values to proportions based on standard length, body depth, body width, head length, or head depth depending on the relevant structure and dimension (Table 1; Casatti, Langeani, Silva, & Castro, 2006; Winemiller, 1991). Each species was assigned to a life history category based on information from the literature to be used in the combined‐traits dataset for calculating functional diversity (Table S1).

**Table 1:** All measured traits, trait codes, and trait definitions

Trait	Dataset	Transformation	Trait definition
Average standard length	Habitat	SL	Maximum standard length from the populations in this study
Head length	Habitat	HEAD_L/SL	Distance from the tip of the jaw to the posterior edge of the operculum
Head depth	Habitat	HEAD_D/BOD_D	Vertical distance from dorsum to ventrum passing through the pupil
Oral gape	Diet	GAPE/BOD_D	Vertical distance measured inside of fully open mouth at tallest point
Mouth position	Both	MOUTH_P	The angle between an imaginary line connecting the tips of the open jaws and an imaginary line running between the center of the pupil and the posterior‐most vertebra (e.g., 90 representing a terminal mouth)
Eye position	Both	EYE_POS/HEAD_D	Vertical distance from the ventral pigmented region to the ventrum
Eye diameter	Both	EYE_D/HEAD_D	Vertical distance from eye margin to eye margin
Snout length	Habitat	SNT_L/HEAD_L	Distance from the posterior pigmented region of the eye to the tip of the upper jaw with mouth shut
Snout protrusion	Diet	SNT_PR/HEAD_L	Additional distance from the posterior pigmented region to the tip of the upper jaw with mouth fully open and extended
Body depth	Habitat	BOD_D/SL	Maximum vertical distance from dorsum to ventrum
Body width	Habitat	BOD_W/SL	Maximum horizontal distance from side to side
Caudal peduncle length	Habitat	PED_L/SL	Distance from the posterior proximal margin of the anal fin to the caudal margin of the ultimate vertebra
Caudal peduncle depth	Habitat	PED_D/BOD_D	Minimum vertical distance from dorsum to ventrum of caudal peduncle
Caudal peduncle width	Habitat	PED_W/BOD_W	Horizontal width of the caudal peduncle at mid‐length
Dorsal fin length	Habitat	DORS_L/SL	Distance from the anterior proximal margin to the posterior proximal margin of the dorsal fin
Dorsal fin height	Habitat	DORS_HT/SL	Maximum distance from the proximal to distal margin of the dorsal fin (excluding filaments)
Anal fin length	Habitat	ANAL_L/SL	Distance from the anterior proximal margin to the posterior proximal margin of the anal fin
Anal fin height	Habitat	ANAL_HT/SL	Maximum distance from proximal to distal margin of the anal fin
Caudal fin depth	Habitat	CAUD_D/SL	Maximum vertical distance across the fully spread caudal fin
Caudal fin length	Habitat	CAUD_L/SL	Maximum distance from proximal to distal margin of the caudal fin (excluding filaments)
Pectoral fin length	Habitat	PEC_L/SL	Maximum distance from proximal to distal margin of pectoral fin
Pelvic fin length	Habitat	PELV_L/SL	Maximum distance from the proximal to distal margin of the pelvic fin
Gut length	Diet	GUT_L/SL	Length of gut from the beginning of the esophagus to the anus (extended without stretching)
Gill raker	Diet	RAKER	Coded as 0 for absent, 1 for short, blunt, or toothlike, 2 for intermediate or long and sparse, and 3 for long and comb‐like
Tooth shape	Diet	TOO_S	Coded as 0 for absent, 1 for unicuspid (rasping), 2 for multicuspid (crushing), 3 for short conical (grasping), 4 for long conical (piercing), and 5 for triangular serrated (shearing)

## 3 Statistical methods

### 3.1 Metrics of functional diversity

Analysis of diverse traits can provide an integrated assessment of assemblage functional structure (Violle et al., 2007). However, if contrasting assemblage processes act on different niche dimensions, opposing trait patterns could mask each other and produce a neutral pattern of trait dispersion (Swenson & Enquist, 2009; Trisos et al., 2014). Analysis of traits that are clearly associated with a given niche dimension may facilitate inference of niche‐based assembly processes, whereas combining traits from multiple niche dimensions may give an integrated overview of assemblage structure (Trisos et al., 2014; Fitzgerald, Winemiller, Pérez, & Sousa, 2017a). Therefore, three sets of functional traits were analyzed: (a) traits associated with habitat use (20 habitat traits), (b) traits associated with food acquisition (8 feeding traits), and (c) both of these trait sets combined combination of habitat traits and diet traits, with and life history categories (26 combined traits). Three standard indices were used to determine functional diversity for each trait grouping: Rao's quadratic entropy (RaoQ), functional richness (FRic), and mean nearest neighbor distance (MNND). These indexes are recommended as robust measures of trait overdispersion (NMMD and RaoQ) and underdispersion (FRic and RaoQ; Aiba et al., 2013; Botta‐Dukát & Czúcz, 2016). The dbFD function from the FD package was used to calculate RaoQ and FRic multitrait metrics (Laliberté, Legendre, & Shipley, 2014) weighted by abundance. The *picante* package in R was used to calculate the MNND metric (Kembel et al., 2010). Because the number of trait axes must be less than the number of species in each sample point, only the first two axes of the principal coordinates analysis (PCoA) were used in the dbFD function. Due to their extreme morphology, a few anguilliform (eel‐like) species were removed before calculating diversity metrics; their inclusion produced strongly skewed gradients and assemblage ordinations that widely separated anguilliform fishes from all other species, with the latter and much larger group tightly clustered within morphospace.

### 3.2 Dispersion, scale, and null models

Null models were used to test whether the observed functional metrics were significantly different from random. Local assemblages and regional species pools were evaluated at two spatial scales: (a) microhabitats, with the corresponding stream reach serving as the regional species pool, and (b) stream reaches, with the collective list of species captured from streams of the corresponding region serving as the regional species pool. For each FD metric, null model, and location, the standard effect size (SES) was calculated as (mean_observed_ − mean_simulated_)/*SD*_simulated_. Standard effect size values greater than 0 signify trait overdispersion, whereas SES values less than 0 demonstrate trait clustering. An alpha value of 0.1 was used in this study. The observed value was determined to be significantly different from random when the observed FD index value ranked higher than 950th or lower than 50th out of a 1,000 when compared to the ranked null FD index values (*p* value = observed rank/runs + 1).

Two null models were used to test whether the observed dispersion indexes differ from random. Null models differ in their ability to discern assemblage mechanisms, and a family of null models should be used to identify different assemblage processes (Chalmandrier et al., 2013; Götzenberger et al., 2016). To test for community assembly mechanisms, we used two commonly recommended null models, independent‐swap and taxon‐label (Cornwell & Ackerly, 2009; Fitzgerald, Winemiller, Sabaj Pérez, & Sousa, 2017b; Gotelli, 2000; Götzenberger et al., 2016; Lavender, Schamp, & Lamb, 2016). The independent‐swap model randomizes species abundance matrix while preserving the species richness and species occurrence at sites and is thought to be more appropriate for short‐term data (Gotelli, 2000). The taxon‐label model shuffles species names in the trait dataset without constraint and has been recommended for detecting limiting similarity (Götzenberger et al., 2016). The likelihood of detecting competitive exclusion may be strongly affected by the regional species pool selected (Götzenberger et al., 2016; Swenson, Enquist, Pither, Thompson, & Zimmerman, 2006; Troia & Gido, 2015). Local species pools, a subset of the regional species pool, would inevitably be less functionally diverse than the regional species pool, especially if environmental filtering is acting on the local species pool. In this case, overdispersion may not be identified because the local species pool is already underdispersed relative to the regional species pool, even if niche segregation is occurring in this local species pool. Our regional species pools were phylogenetically diverse, spanning several taxonomic orders with little functional redundancy, possibly reducing the likelihood of detecting overdispersion (Table S1). Therefore, we also use the taxon‐label model to detect limiting similarity using groupings of similar habitats within a given region as the regional species pool, and sample points of the same habitat grouping as the local species pool, accounting for any previous environmental filtering and reduction in functional diversity. Habitat groupings were made according to criteria and methods reported in Bower, Saenz, Winemiller, (Inpress). These models were run using the RandomizeMatrix and taxaShuffle functions in the *picante* package in R (Kembel et al., 2010).

For the phylogenetic analyses, we acquired a previously published, time‐calibrated tree by Rabosky et al. (2018), and then trimmed the tree to include only species collected in our study. Because some species in our study were not included in this tree, we followed the protocol of Beaulieu, Ree, Cavender‐Bares, Weiblen, and Donoghue (2012) and inserted these species in place of closely related taxa to create a tree that included all species in our study (Table S1). To assess the phylogenetic structure of fish assemblages, the net relatedness index (NRI) and nearest taxon index (NTI) were used (Brunbjerg et al., 2014; Webb, Ackerly, McPeek, & Donoghue, 2002). Net relatedness index and NTI were calculated as.

$$(\left(r_{\text{obs}}-r_{\text{null}}\right)/SD_{\text{null}})*-1,$$

where in *r* is the mean pairwise distance (MPD) when calculating NRI, and *r* is the mean nearest taxon distance (MNTD) when calculating NTI. The null models for *r*_null_ were created by randomly swapping the tips of the phylogeny 999 times while weighting by species abundance using the taxa‐labels null model in the R package picante (Kembel et al., 2010). Negative values of NRI and NTI indicate phylogenetic overdispersion, with co‐occurring species being less closely related than expected at random, and positive values show phylogenetic clustering, whereby co‐occurring species are more closely related than expected at random. Both NRI and NTI were calculated for fish assemblages at the microhabitat scale with the matching stream reach serving as the regional species pool, and then again at the stream‐reach scale with the corresponding region serving as the regional species pool. Random intercept linear mixed models and general linear mixed models with a gamma distribution were used to test the correlation between habitat variables and FRic, NMMD, RaoQ, MPD, and MNTD values. Model type was selected based on how well the data fit the model assumptions. In these models, functional diversity metrics were the dependent variable, with water velocity, water depth, and substrate complexity as independent variables and region and sampling site as random factors. Habitat variables and functional metrics were log‐transformed to meet the model assumptions. The “ANOVA” function from the *car* package in R was used to test whether each habitat variable significantly influenced the dependent variable.

## 4 RESULTS

Overall, 230 fish species were collected and analyzed: 21 from Belize, 53 from Brazil, 26 from Benin, 67 from Cambodia, and 63 from the USA. The Cambodia region had the most families represented (20), followed by Brazil (19), Benin (17), USA (12), and Belize (9) (Table S1). The average species per microhabitat for each region was as follows: Cambodia, 5.79 species; Benin, 4.02; USA, 3.88; Belize, 3.82; and Brazil 3.51.

### 4.1 Patterns of functional diversity at the microhabitat scale

**Figure 3:**
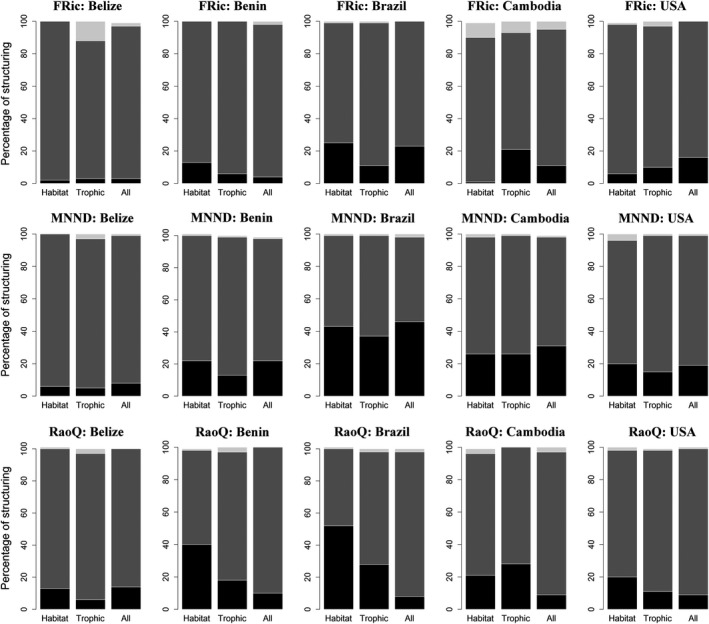
Functional trait diversity for each region based on taxon‐label model and all three metrics: FRic, MNND, and RaoQ. Proportions of significantly overdispersed (light gray), underdispersed (black), and randomly (dark gray) structured local assemblages at the microhabitat scale using the corresponding stream reach as the regional species pool

Evidence for both over‐ and underdispersion of traits associated with habitat use was found at the local scale, where fish collected from microhabitat units were defined as local assemblages and the collective fish sample from the corresponding stream reach defined the regional species pool (Figures 2 and Figure S1). Significant underdispersion was detected more often than overdispersion for all functional diversity metrics, trait groupings, null models, and regions, with two exceptions (Figures 3 and Figure S1). First, more overdispersion than underdispersion was found for Belize fish assemblages when the analysis used the trophic‐traits dataset and FRic metric. Second, Cambodia fish assemblages displayed more overdispersion than underdispersion using the habitat‐traits dataset and FRic metric (Figures 3 and Figure S1). Highest percentages of local (i.e., microhabitat) assemblages that were overdispersed were found in Belize and Cambodia (Figure 3 and Figure S1–S3). Brazil had the greatest percentage of local assemblages that were underdispersed, followed by Cambodia and Benin assemblages (Figure 3), whereas Belize assemblages tended to have lowest percentages of underdispersed local assemblages (Figure 3).

### 4.2 Patterns of functional diversity at the stream‐reach scale

When stream reach was used to define local species assemblages, there were more instances of underdispersion than overdispersion in every region (Tables [Link], [Link], [Link]). Assemblages in Belize showed significant underdispersion across all functional trait metrics. However, using the feeding‐traits dataset and RaoQ metric, two instances of overdispersion were observed using the MNND metric and independent‐swap model (Table S2). In both Benin and Brazil, local assemblages at the reach scale were found to be underdispersed for all functional diversity metrics, with RaoQ showing the most underdispersion (Tables [Link], [Link], [Link]). A single instance of overdispersion was found for both Benin and Brazil when the analysis was for the combined‐traits dataset using the taxon‐label model and FRic metric (Table S1). Underdispersion was observed for stream‐reach assemblages in Cambodia when the analysis was based on the combined‐traits dataset for all functional diversity metrics and null models (Tables [Link], [Link], [Link]), the only exception being the RaoQ metric analyzed with the taxon‐label model. For US assemblages, underdispersion only resulted from analyses using the RaoQ metric (Tables [Link], [Link], [Link]).

### 4.3 Phylogenetic dispersion of local assemblages at the microhabitat scale

**Figure 4:**
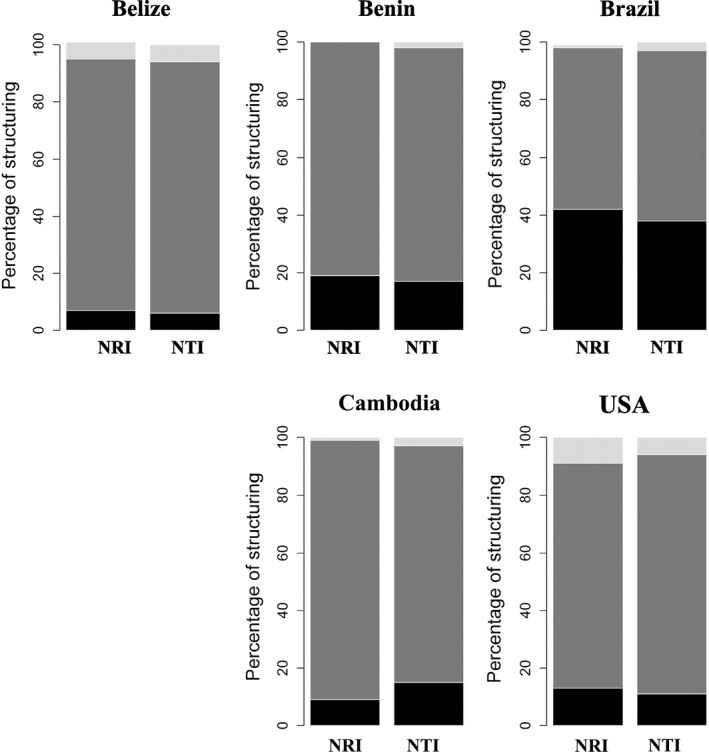
Phylogenetic diversity for each region based on taxon‐label model and both metrics: NTI and NRI. Proportions of significantly overdispersed (light gray), underdispersed (black), and randomly (dark gray) structured local assemblages at the microhabitat scale using the corresponding stream reach as the regional species pool

With local assemblages defined at the scale of the stream reach, stream fish assemblages in Brazil tended be more phylogenetically related than expected by chance, with 38% and 42% of local assemblages being underdispersed for NTI and NRI metrics of phylogenetic distance (Figure 4). Between 9% and 19% local assemblages (reach scale) in each of the other four regions were underdispersed at the microhabitat scale using both metrics of phylogenetic distance (Figure 4). Belize and US assemblages had highest percentages of phylogenetic overdispersion, between 6% and 9% for both metrics (Figure 4). In the other regions, phylogenetic overdispersion was found for less than 3% of microhabitat sites based on either metric.

### 4.4 Phylogenetic dispersion of local assemblages at the reach scale

None of the Brazilian assemblages at the reach scale were found to be significantly over‐ or underdispersed when the analysis was based on NRI or NTI. For Belize and Cambodia, none of the local assemblages at the reach scale revealed significant phylogenetic over‐ or underdispersion based on either metric of phylogenetic distance. Benin and USA each had one instance of significant underdispersion based on analysis with the NRI. In addition, one stream reach in the Benin region was found to be phylogenetically underdispersed using the NTI. Significant overdispersion was observed for two US assemblages based on NRI, and for one US assemblage based on NTI.

### 4.5 Diversity patterns along environmental gradients

**Figure 5:**
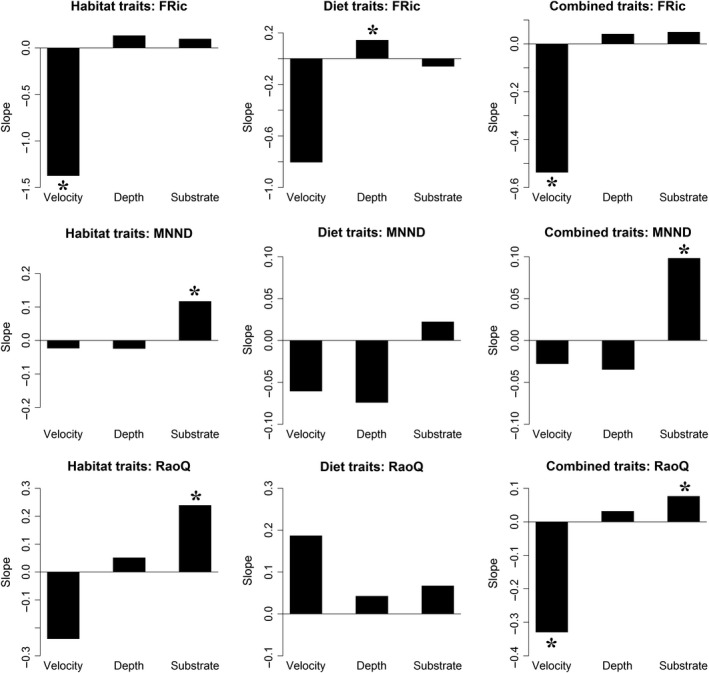
The slopes from the mixed models testing for a relationship between habitat variables (water velocity, water depth, and substrate complexity) and functional trait metrics (FRic, MNND, and RaoQ) using habitat‐traits, feeding‐traits, and combined‐traits datasets. Asterisk denotes significance (*p* value < .05)

Water velocity, depth, and substrate complexity were correlated with functional diversity metrics for both the habitat‐use and combined‐traits datasets (Figure 5). For the habitat‐traits dataset and combined‐traits dataset, the FRic metric was negatively correlated with water velocity. The RaoQ metric was also negatively correlated with water velocity when the analysis was performed on the combined‐traits dataset (Figure 5). However, the FRic metric was found to have a positive relationship with water depth when using the diet‐traits dataset (Figure 5). For the habitat‐use and combined‐traits datasets, substrate was shown to have a positive relationship with MNND and RaoQ metrics (Figure 5). A marginally significant negative relationship was found between water velocity and MNTD (Slope −0.337, *p* value = .073), and no significant relationship was detected between the MPD and any habitat variable.

## 5 DISCUSSION

Results from this study imply that environmental filtering and, to a lesser extent, species interactions structure fish assemblages in small, low‐gradient streams in five zoogeographic regions. Habitat‐traits, feeding‐traits, and combined‐traits datasets showed more instances of underdispersion than overdispersion regardless of spatial scale and regional species pool. These results generally support the paradigm that environmental filtering has a greater influence on fish assemblage structure than species interactions that limit interspecific similarity (Córdova‐Tapia et al., 2018; Mouillot et al., 2007; Troia & Gido, 2015). In addition, we found reduced functional diversity in microhabitats with more stressful environmental conditions, such as high water velocity, shallow water depth, and homogeneous substrates lacking structural complexity, which lends support for the stress‐dominance hypothesis (Coyle et al., 2014; Ramm et al., 2018; Swenson & Enquist, 2007; Weiher & Keddy, 1995).

### 5.1 Patterns of trait dispersion

#### 5.1.1 Underdispersion

Defining spatial scale and sampling grain size is critical for understanding how community assembly processes influence species co‐occurrence (Weiher et al., 2011; Trisos et al., 2014). In contrast to our first expectation, we did not find a shift from underdispersion of traits at the reach scale to overdispersion of traits at the microhabitat scale. Instead, underdispersion was common at both spatial scales and consistent across zoogeographic regions, even when using similar habitat groupings as the regional species pool (Figure 3; Figures S1, S3; Tables [Link], [Link], [Link]). This suggests that environmental filtering is more important than limiting similarity for fishes inhabiting small, low‐gradient streams. However, the magnitude of trait dispersion patterns depended on the functional metric, null model, and types of traits employed in the analysis. In our study, fish assemblages in Brazil and Cambodia revealed strongest patterns of trait underdispersion. This finding may be due to the high functional diversity of stream fishes in these regions, which might increase the likelihood of producing significant underdispersion. The amount of trait variation from a regional species pool that is assembled into local assemblage likely will be proportionally small when the regional species pool has high functional diversity.

#### 5.1.2 Overdispersion

In this study, limited evidence of overdispersion suggests that interspecific competition and other species interactions play a secondary and perhaps minor role in structuring stream fish assemblages. The low incidence of overdispersion at the local scale was unexpected for tropical fishes that have much higher functional trait diversity compared to temperate fishes (Montaña et al., 2014; Schemske, Mittelbach, Cornell, Sobel, & Roy, 2009; Winemiller, 1991), with the exception of several fish assemblages in Cambodia and Belize that revealed higher instances of overdispersion. Agreeing with our expectation that higher instances of limiting similarity should be detected when the analysis was performed using traits associated with feeding, overdispersion was detected for both Cambodia and Belize, suggesting competition for food resources (Trisos et al., 2014). Yet, this trend was not seen for the other regions. Overdispersion was also detected for habitat‐traits and combined‐traits datasets in Cambodia region, which may be due to interspecific partitioning of microhabitats. However, assemblages in Cambodia generally had more species per microhabitat than the other regions, thus increasing the potential for interspecific interactions. Slightly larger streams were sampled in Cambodia, which could have contributed to more species per microhabitat. The size of the microhabitats (areas of relatively homogeneous depth, current velocity, substrate composition, and in‐channel cover) within a stream tended to increase with stream size. The average species per microhabitat was approximately four in the other regions, with many microhabitats having more than five species; yet, evidence for overdispersion at the microhabitat scale was very limited. The low number of species per microhabitat also may have contributed to the high percentage of nonsignificant dispersion values. However, this is unlikely, because linear regressions did not yield any significant relationships between *p* values for trait dispersion and number of species in microhabitat samples. A more likely explanation is that competitive exclusion influenced by traits associated with resource acquisition only occurs when resources are limiting. Habitat disturbance from periodic high flow events in small streams may reduce fish populations below carry capacity (Harvey, 1987; Poff & Allan, 1995; Resh et al., 1988), thus negating resource competition and introducing a stochastic component to population and community dynamics (Chase, 2007; Resh et al., 1988).

#### 5.1.3 Random dispersion

Although significant over‐ or underdispersion was found for various microhabitats, a majority of trait dispersion values were no different from random, implying stochastic factors or opposing assembly mechanisms influenced stream fish assemblages. Contrasting assemblage mechanisms may mask each other producing a net neutral pattern of trait dispersion (Swenson & Enquist, 2009; Trisos et al., 2014). We attempted to deal with this issue by grouping traits according to two different niche dimensions (feeding behavior vs. locomotion/habitat use), yet certain traits may have a one‐to‐many relationship of form and function (Hulsey & Wainwright, 2002). For example, the sucker‐like mouth of armored catfish (Loricariidae) is used to scrape algae and detritus from hard substrates but can also be used for attachment to substrates in order to maintain position in strong currents (Pagotto, Goulart, Oliveira, & Yamamura, 2011). In this case, attributes of the mouth could be associated with both feeding and habitat use. This issue likely is more challenging when phylogenetic diversity and functional diversity of datasets are expanded. In addition, the signal of niche‐based processes may not be detected if traits other than the ones used in this study are the ones influenced by these processes. The high mobility of fishes may increase the potential influence of stochastic aspects of dispersal. Highly mobile organisms may move briefly into and out of areas of strong competition or environmental stress, so that the assemblage patterns appear stochastic when sampling is based on a limited time interval and area (Gomez, Bravo, Brumfield, Tello, & Cadena, 2010; Harmon‐Threatt & Ackerly, 2013; Weiher et al., 2011).

### 5.2 Phylogenetic diversity patterns

**Figure 6:**
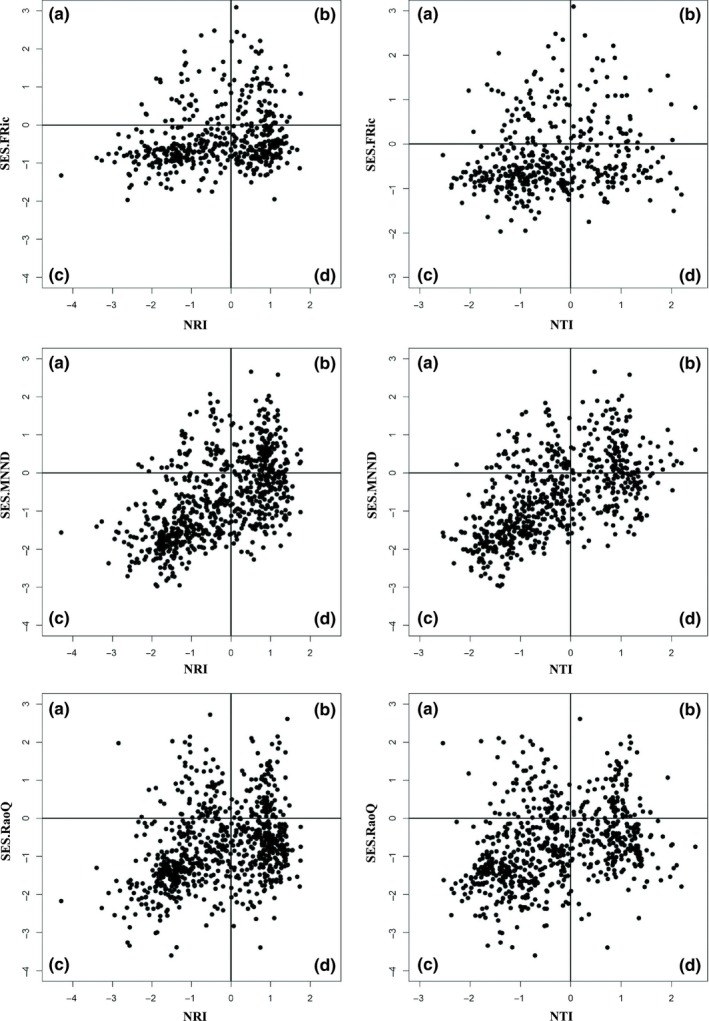
Standardized effect size (SES) for FRic, MNND, or RaoQ plotted against NTI or NRI based on the taxon‐label model using habitat traits. Lines divide plot into quadrats. Quadrat (a) suggests morphological divergence and niche segregation of related species; (b) morphological divergence and niche segregation of unrelated species; (c) morphological underdispersion of related species due to stabilizing selection or niche conservatism; (d) morphological convergence of unrelated species reflecting habitat filtering of convergent forms

Phylogenetic underdispersion was more prevalent among assemblages from Benin, Brazil, and Cambodia, with species co‐occurring within microhabitats more closely related than expect by chance. In contrast, the percentage of assemblages showing overdispersion was similar to those revealing underdispersion in Belize and USA. Regional differences in phylogenetic dispersion patterns likely are associated with variation in number of evolutionary lineages and assemblage composition. In addition, the evolutionary age of habitat types may influence phylogenetic dispersion patterns (Gerhold et al., 2015). Significant overdispersion in Belize and USA assemblages indicates that unrelated species occupied the same microhabitat. Previous studies have suggested that this pattern demonstrates limiting similarity (Cavender‐Bares, Kozak, Fine, & Kembel, 2009; Swenson & Enquist, 2007). However, phylogenetic dispersion should be interpreted with caution and not may be suitable for detecting community assembly processes, but instead can give insights into assemblage evolution (Gerhold et al., 2015). Significant functional underdispersion coupled with nonsignificant dispersion or significant overdispersion of phylogenetic data may reflect habitat filtering of convergent forms, implying that unrelated species with similar traits responded to similar environments in a congruent manner. This pattern of functional underdispersion coupled with nonsignificant phylogenetic dispersion was observed in our study (Figures 6 and Figure [Link], [Link]). However, environmental filters can lead to phylogenetic underdispersion if a clade has similar traits and environmental tolerances. Many cases where trait and phylogenetic underdispersion occurred simultaneously were identified in the current study (Figures 6 and Figure [Link], [Link]). Here, we infer that closely related species in similar microhabitats have converged on similar traits via stabilizing selection (Gerhold et al., 2015). Another explanation for this may be niche conservatism, whereby species retain ancestral niches over time (Harvey & Pagel, 1991; Wiens et al., 2010; Wiens & Graham, 2005), suggesting that macroevolutionary processes influence local assemblage structure.

### 5.3 Functional diversity along environmental gradients

The stress‐dominance hypothesis proposes that stressful environments exclude species with unsuitable traits, resulting in local assemblages with high trait similarity (Weiher & Keddy, 1995). In stream fishes, functional diversity metrics were related to water depth, substrate complexity, and water velocity in a manner consistent with the stress‐dominance hypothesis. This pattern was fairly congruent across regions (Table S5; Bower & Winemiller, 2019). FRic was inversely associated with water velocity, suggesting that requirements for coping with hydraulic drag restrict assemblage trait space in stream microhabitats with fast flows. Several studies have found significant relationships between water velocity and fish assemblage structure in streams (Bower & Piller, 2015; Haas et al., 2015; Lamouroux et al., 2002; Willis et al., 2005). The energetic cost of occupying a microhabitat with high flow velocity restricts functional diversity (Webb, 1984, 1988). In our study, fish functional diversity increased with water depth, a finding consistent with other studies (Carvalho & Tejerina‐Garro, 2015; Leitão et al., 2018). We captured fishes from water as shallow as 3 cm where many fishes would be excluded based on body size alone. Moreover, predation threat from birds is greater in shallow habitats (Bancroft, Gawlik, & Rutchey, 2002; Keppeler, Cruz, Dalponti, & Mormul, 2016), further restricting fish functional diversity.

In our study, functional diversity metrics were positively associated with substrate complexity. Structural complexity has been shown to reduce both abiotic and biotic stresses by providing a refuge from harsh environmental conditions and predators (Kovalenko et al., 2012). Structural complexity in streams often is associated with higher species richness and functional diversity (Ceneviva‐Bastos, Montaña, Schalk, Camargo, & Casatti, 2017; Emslie, Cheal, & Johns, 2014; Kovalenko et al., 2012; Mouillot, Graham, Villéger, Mason, & Bellwood, 2013). Our results overall indicated the dominant influence of environmental filtering and were consistent with the stress‐dominance hypothesis.

## 6 CONCLUSIONS

Relationships between habitat variables and functional diversity metrics indicate that environmental filtering is an important mechanism of community assembly for stream fishes in several regions of the world. With the exception of Belize and Cambodia, limiting similarity does not appear to exert a strong influence on the structure of stream fish assemblages at the two spatial scales of analysis employed here, which contrasts with our expectations. However, caution is warranted when interpreting trait dispersion patterns (Mayfield & Levine, 2010). Other assembly mechanisms, such as facilitation, can also produce nonrandom patterns of trait dispersion (Cavender‐Bares et al., 2009). For example, benthivorous suckers (Catostomidae) can facilitate feeding success of other fishes when they dislodge benthic invertebrates from sediments (Ross & Brenneman, 2001). Manipulative experiments are needed to improve understanding of how traits affect performance and influence the structure and functions of local species assemblages. We found limited evidence of trait overdispersion, and future research should examine traits with different functions and species assemblages spanning broader environmental gradients in space and time. Trait datasets could be compiled to examine patterns for other niche dimensions, including life history, defense, and physiology/metabolism (Winemiller, Fitzgerald, Bower, & Pianka, 2015). For example, Troia and Gido (2015) found that underdispersion of life history traits increased from downstream to headwaters. Our findings suggest that the environmental filtering was the most important mechanism of community assembly for fishes inhabiting small streams in five zoogeographic regions. Water velocity, water depth, and substrate complexity seem to be particularly influential in restricting fish occupation of certain microhabitats. We found a high incidence of functional underdispersion coupled with phylogenetic underdispersion that could reflect phylogenetic niche conservation or stabilizing selection. Our findings suggest that local fish assemblages in small streams worldwide are most strongly influenced by environmental filtering, with weaker effects from species interactions and stochastic processes associated with dispersal.

## CONFLICT OF INTEREST

The authors have no conflicts of interest to declare.

## AUTHOR CONTRIBUTIONS

LMB and KOW conceived the ideas. LMB collected data, analyzed data and wrote manuscript. KOW contributed critically to the draft and gave final approval for publication.

## Data Availability

Trait, species abundance, and environmental data have been uploaded to Dryad. https://doi.org/10.5061/dryad.n53sh18.

## References

[ece35823-bib-0001] Aiba, M. , Katabuchi, M. , Takafumi, H. , Matsuzaki, S. I. S. , Sasaki, T. , & Hiura, T. (2013). Robustness of trait distribution metrics for community assembly studies under the uncertainties of assembly processes. Ecology, 94(12), 2873–2885. 10.1890/13-0269.1 24597232

[ece35823-bib-0002] Algar, A. C. , Kerr, J. T. , & Currie, D. J. (2011). Quantifying the importance of regional and local filters for community trait structure in tropical and temperate zones. Ecology, 92(4), 903–914. 10.1890/10-0606.1 21661553

[ece35823-bib-0003] Bancroft, G. T. , Gawlik, D. E. , & Rutchey, K. (2002). Distribution of wading birds relative to vegetation and water depths in the northern Everglades of Florida, USA. Waterbirds, 25(3), 265–278. 10.1675/1524-4695(2002)025[0265:DOWBRT]2.0.CO;2

[ece35823-bib-0004] Bartholomew, A. , Diaz, R. J. , & Cicchetti, G. (2000). New dimensionless indices of structural habitat complexity: Predicted and actual effects on a predator^1^s foraging success. Marine Ecology Progress Series, 206, 45–58.

[ece35823-bib-0005] Beaulieu, J. M. , Ree, R. H. , Cavender‐Bares, J. , Weiblen, G. D. , & Donoghue, M. J. (2012). Synthesizing phylogenetic knowledge for ecological research. Ecology, 93(sp8), S4–S13. 10.1890/11-0638.1

[ece35823-bib-0006] Blanchet, S. , Helmus, M. R. , Brosse, S. , & Grenouillet, G. (2014). Regional vs local drivers of phylogenetic and species diversity in stream fish communities. Freshwater Biology, 59(3), 450–462.

[ece35823-bib-0007] Botta‐Dukát, Z. , & Czúcz, B. (2016). Testing the ability of functional diversity indices to detect trait convergence and divergence using individual‐based simulation. Methods in Ecology and Evolution, 7(1), 114–126. 10.1111/2041-210X.12450

[ece35823-bib-0008] Bower, L. M. , & Piller, K. R. (2015). Shaping up: A geometric morphometric approach to assemblage ecomorphology. Journal of Fish Biology, 87(3), 691–714. 10.1111/jfb.12752 26268468

[ece35823-bib-0009] Bower, L. M. , Saenz, D. E. , Winemiller, K. O. Widespread convergence in stream fishes. Ecology Letters. Inpress.

[ece35823-bib-0010] Bower, L. M. , & Winemiller, K. O. (2019). Fish assemblage convergence along stream environmental gradients: An intercontinental analysis. Ecography, 42(10), 1691–1702. 10.1111/ecog.04690

[ece35823-bib-0011] Brooker, R. W. , Callaway, R. M. , Cavieres, L. A. , Kikvidze, Z. , Lortie, C. J. , Michalet, R. , … Whitham, T. G. (2009). Don't diss integration: A comment on Ricklefs's disintegrating communities. The American Naturalist, 174(6), 919–927. 10.1086/648058 19860539

[ece35823-bib-0012] Brown, W. L. , & Wilson, E. O. (1956). Character Displacement. Systematic Zoology, 5(2), 49–64. 10.2307/2411924

[ece35823-bib-0013] Brunbjerg, A. K. , Cavender‐Bares, J. , Eiserhardt, W. L. , Ejrnaes, R. , Aarssen, L. W. , Buckley, H. L. , … Svenning, J.‐C. (2014). Multi‐scale phylogenetic structure in coastal dune plant communities across the globe. Journal of Plant Ecology, 7(2), 101–114. 10.1093/jpe/rtt069

[ece35823-bib-0014] Carvalho, R. A. , & Tejerina‐Garro, F. L. (2015). Environmental and spatial processes: What controls the functional structure of fish assemblages in tropical rivers and headwater streams? Ecology of Freshwater Fish, 24(2), 317–328. 10.1111/eff.12152

[ece35823-bib-0015] Casatti, L. , Langeani, F. , Silva, A. M. , & Castro, R. M. C. (2006). Stream fish, water and habitat quality in a pasture dominated basin, southeastern Brazil. Brazilian Journal of Biology, 66(2B), 681–696. 10.1590/S1519-69842006000400012 16906300

[ece35823-bib-0016] Cavender‐Bares, J. , Kozak, K. H. , Fine, P. V. , & Kembel, S. W. (2009). The merging of community ecology and phylogenetic biology. Ecology Letters, 12(7), 693–715. 10.1111/j.1461-0248.2009.01314.x 19473217

[ece35823-bib-0017] Ceneviva‐Bastos, M. , Montaña, C. G. , Schalk, C. M. , Camargo, P. B. , & Casatti, L. (2017). Responses of aquatic food webs to the addition of structural complexity and basal resource diversity in degraded Neotropical streams. Austral Ecology, 42(8), 908–919. 10.1111/aec.12518

[ece35823-bib-0018] Chalmandrier, L. , Münkemüller, T. , Gallien, L. , De Bello, F. , Mazel, F. , Lavergne, S. , & Thuiller, W. (2013). A family of null models to distinguish between environmental filtering and biotic interactions in functional diversity patterns. Journal of Vegetation Science, 24(5), 853–864. 10.1111/jvs.12031 24791143 PMC4003529

[ece35823-bib-0019] Chase, J. M. (2007). Drought mediates the importance of stochastic community assembly. Proceedings of the National Academy of Sciences of the United States of America, 104(44), 17430–17434. 10.1073/pnas.0704350104 17942690 PMC2077273

[ece35823-bib-0020] Chase, J. M. , & Myers, J. A. (2011). Disentangling the importance of ecological niches from stochastic processes across scales. Philosophical Transactions of the Royal Society B: Biological Sciences, 366(1576), 2351–2363. 10.1098/rstb.2011.0063 PMC3130433 21768151

[ece35823-bib-0021] Córdova‐Tapia, F. , Hernández‐Marroquín, V. , & Zambrano, L. (2018). The role of environmental filtering in the functional structure of fish communities in tropical wetlands. Ecology of Freshwater Fish, 27(2), 522–532. 10.1111/eff.12366

[ece35823-bib-0022] Cornwell, W. K. , & Ackerly, D. D. (2009). Community assembly and shifts in plant trait distributions across an environmental gradient in coastal California. Ecological Monographs, 79(1), 109–126. 10.1890/07-1134.1

[ece35823-bib-0023] Coyle, J. R. , Halliday, F. W. , Lopez, B. E. , Palmquist, K. A. , Wilfahrt, P. A. , & Hurlbert, A. H. (2014). Using trait and phylogenetic diversity to evaluate the generality of the stress‐dominance hypothesis in eastern North American tree communities. Ecography, 37(9), 814–826. 10.1111/ecog.00473

[ece35823-bib-0024] Dimitriadis, C. , Evagelopoulos, A. , & Koutsoubas, D. (2012). Functional diversity and redundancy of soft bottom communities in brackish waters areas: Local vs regional effects. Journal of Experimental Marine Biology and Ecology, 426, 53–59. 10.1016/j.jembe.2012.05.016

[ece35823-bib-0025] Emslie, M. J. , Cheal, A. J. , & Johns, K. A. (2014). Retention of habitat complexity minimizes disassembly of reef fish communities following disturbance: A large‐scale natural experiment. PLoS ONE, 9(8), e105384 10.1371/journal.pone.0105384 25140801 PMC4139330

[ece35823-bib-0026] Fitzgerald, D. B. , Winemiller, K. O. , Pérez, M. H. S. , & Sousa, L. M. (2017a). Using trophic structure to reveal patterns of trait‐based community assembly across niche dimensions. Functional Ecology, 31(5), 1135–1144.

[ece35823-bib-0027] Fitzgerald, D. B. , Winemiller, K. O. , Sabaj Pérez, M. H. , & Sousa, L. M. (2017b). Seasonal changes in the assembly mechanisms structuring tropical fish communities. Ecology, 98(1), 21–31. 10.1002/ecy.1616 27984648

[ece35823-bib-0028] Gatz, A. J. Jr (1979). Ecological morphology of freshwater stream fishes. Tulane Studies in Zoology and Botany, 21(2), 91–124.

[ece35823-bib-0029] Gerhold, P. , Cahill, J. F. , Winter, M. , Bartish, I. V. , & Prinzing, A. (2015). Phylogenetic patterns are not proxies of community assembly mechanisms (they are far better). Functional Ecology, 29(5), 600–614. 10.1111/1365-2435.12425

[ece35823-bib-0030] Gomez, J. P. , Bravo, G. A. , Brumfield, R. T. , Tello, J. G. , & Cadena, C. D. (2010). A phylogenetic approach to disentangling the role of competition and habitat filtering in community assembly of Neotropical forest birds. Journal of Animal Ecology, 79(6), 1181–1192. 10.1111/j.1365-2656.2010.01725.x 20642767

[ece35823-bib-0031] Gotelli, N. J. (2000). Null model analysis of species co‐occurrence patterns. Ecology, 81(9), 2606–2621. 10.1890/0012-9658(2000)081[2606:NMAOSC]2.0.CO;2

[ece35823-bib-0032] Götzenberger, L. , Botta‐Dukát, Z. , Lepš, J. , Pärtel, M. , Zobel, M. , & de Bello, F. (2016). Which randomizations detect convergence and divergence in trait‐based community assembly? A test of commonly used null models. Journal of Vegetation Science, 27(6), 1275–1287. 10.1111/jvs.12452

[ece35823-bib-0033] Götzenberger, L. , de Bello, F. , Bråthen, K. A. , Davison, J. , Dubuis, A. , Guisan, A. , … Zobel, M. (2012). Ecological assembly rules in plant communities—Approaches, patterns and prospects. Biological Reviews, 87(1), 111–127. 10.1111/j.1469-185X.2011.00187.x 21692965

[ece35823-bib-0034] Haas, T. C. , Heins, D. C. , & Blum, M. J. (2015). Predictors of body shape among populations of a stream fish (*Cyprinella venusta*, Cypriniformes: Cyprinidae). Biological Journal of the Linnean Society, 115(4), 842–858.

[ece35823-bib-0035] Harmon‐Threatt, A. N. , & Ackerly, D. D. (2013). Filtering across spatial scales: Phylogeny, biogeography and community structure in bumble bees. PLoS ONE, 8(3), e60446 10.1371/journal.pone.0060446 23544141 PMC3609857

[ece35823-bib-0036] Harvey, B. C. (1987). Susceptibility of young‐of‐the‐year fishes to downstream displacement by flooding. Transactions of the American Fisheries Society, 116(6), 851–855. 10.1577/1548-8659(1987)116<851:SOYFTD>2.0.CO;2

[ece35823-bib-0037] Harvey, P. H. , & Pagel, M. D. (1991). The comparative method in evolutionary biology (Vol. 239). Oxford, UK: Oxford University Press.

[ece35823-bib-0038] Heino, J. , Schmera, D. , & Erős, T. (2013). A macroecological perspective of trait patterns in stream communities. Freshwater Biology, 58(8), 1539–1555.

[ece35823-bib-0039] HilleRisLambers, J. , Adler, P. B. , Harpole, W. S. , Levine, J. M. , & Mayfield, M. M. (2012). Rethinking community assembly through the lens of coexistence theory. Annual Review of Ecology, Evolution, and Systematics, 43, 227–248. 10.1146/annurev-ecolsys-110411-160411

[ece35823-bib-0040] Hoeinghaus, D. J. , Winemiller, K. O. , & Birnbaum, J. S. (2007). Local and regional determinants of stream fish assemblage structure: Inferences based on taxonomic vs. functional groups. Journal of Biogeography, 34(2), 324–338. 10.1111/j.1365-2699.2006.01587.x

[ece35823-bib-0041] Hulsey, C. D. , & Wainwright, P. C. (2002). Projecting mechanics into morphospace: Disparity in the feeding system of labrid fishes. Proceedings of the Royal Society of London. Series B: Biological Sciences, 269(1488), 317–326. 10.1098/rspb.2001.1874 11839201 PMC1690891

[ece35823-bib-0042] Ingram, T. , & Shurin, J. B. (2009). Trait‐based assembly and phylogenetic structure in northeast Pacific rockfish assemblages. Ecology, 90(9), 2444–2453. 10.1890/08-1841.1 19769123

[ece35823-bib-0043] Kembel, S. W. , Cowan, P. D. , Helmus, M. R. , Cornwell, W. K. , Morlon, H. , Ackerly, D. D. , … Webb, C. O. (2010). Picante: R tools for integrating phylogenies and ecology. Bioinformatics, 26(11), 1463–1464. 10.1093/bioinformatics/btq166 20395285

[ece35823-bib-0044] Keppeler, F. W. , Cruz, D. A. , Dalponti, G. , & Mormul, R. P. (2016). The role of deterministic factors and stochasticity on the trophic interactions between birds and fish in temporary floodplain ponds. Hydrobiologia, 773(1), 225–240. 10.1007/s10750-016-2705-y

[ece35823-bib-0045] Kovalenko, K. E. , Thomaz, S. M. , & Warfe, D. M. (2012). Habitat complexity: Approaches and future directions. Hydrobiologia, 685(1), 1–17. 10.1007/s10750-011-0974-z

[ece35823-bib-0046] Kraft, N. J. , Cornwell, W. K. , Webb, C. O. , & Ackerly, D. D. (2007). Trait evolution, community assembly, and the phylogenetic structure of ecological communities. The American Naturalist, 170(2), 271–283. 10.1086/519400 17874377

[ece35823-bib-0047] Laliberté, E. , Legendre, P. , & Shipley, B. (2014). FD: measuring functional diversity from multiple traits, and other tools for functional ecology. R package version 1.0‐12. 10.1890/08-2244.1 20380219

[ece35823-bib-0048] Lamouroux, N. , Poff, N. L. , & Angermeier, P. L. (2002). Intercontinental convergence of stream fish community traits along geomorphic and hydraulic gradients. Ecology, 83(7), 1792–1807. 10.1890/0012-9658(2002)083[1792:ICOSFC]2.0.CO;2

[ece35823-bib-0049] Lavender, T. M. , Schamp, B. S. , & Lamb, E. G. (2016). The influence of matrix size on statistical properties of co‐occurrence and limiting similarity null models. PLoS ONE, 11(3), e0151146 10.1371/journal.pone.0151146 26942941 PMC4778770

[ece35823-bib-0050] Leitão, R. P. , Zuanon, J. , Mouillot, D. , Leal, C. G. , Hughes, R. M. , Kaufmann, P. R. , … Gardner, T. A. (2018). Disentangling the pathways of land use impacts on the functional structure of fish assemblages in Amazon streams. Ecography, 41(1), 219–232. 10.1111/ecog.02845 29910537 PMC5998685

[ece35823-bib-0051] Levin, S. A. (1992). The problem of pattern and scale in ecology: The Robert H. MacArthur Award Lecture. Ecology, 73(6), 1943–1967.

[ece35823-bib-0052] Lujan, N. K. , & Conway, K. W. (2015). Life in the fast lane: A review of rheophily in freshwater fishes In Extremophile Fishes (pp. 107–136). Cham, Switzerland: Springer.

[ece35823-bib-0053] MacArthur, R. , & Levins, R. (1967). The limiting similarity, convergence, and divergence of coexisting species. The American Naturalist, 101(921), 377–385. 10.1086/282505

[ece35823-bib-0054] Mayfield, M. M. , & Levine, J. M. (2010). Opposing effects of competitive exclusion on the phylogenetic structure of communities. Ecology Letters, 13(9), 1085–1093. 10.1111/j.1461-0248.2010.01509.x 20576030

[ece35823-bib-0055] McGill, B. J. , Enquist, B. J. , Weiher, E. , & Westoby, M. (2006). Rebuilding community ecology from functional traits. Trends in Ecology and Evolution, 21(4), 178–185. 16701083 10.1016/j.tree.2006.02.002

[ece35823-bib-0056] Montaña, C. G. , Winemiller, K. O. , & Sutton, A. (2014). Intercontinental comparison of fish ecomorphology: Null model tests of community assembly at the patch scale in rivers. Ecological Monographs, 84(1), 91–107. 10.1890/13-0708.1

[ece35823-bib-0057] Mouchet, M. A. , Burns, M. D. , Garcia, A. M. , Vieira, J. P. , & Mouillot, D. (2013). Invariant scaling relationship between functional dissimilarity and co‐occurrence in fish assemblages of the Patos Lagoon estuary (Brazil): Environmental filtering consistently overshadows competitive exclusion. Oikos, 122(2), 247–257. 10.1111/j.1600-0706.2012.20411.x

[ece35823-bib-0058] Mouillot, D. , Dumay, O. , & Tomasini, J. A. (2007). Limiting similarity, niche filtering and functional diversity in coastal lagoon fish communities. Estuarine, Coastal and Shelf Science, 71(3–4), 443–456. 10.1016/j.ecss.2006.08.022

[ece35823-bib-0059] Mouillot, D. , Graham, N. A. , Villéger, S. , Mason, N. W. , & Bellwood, D. R. (2013). A functional approach reveals community responses to disturbances. Trends in Ecology and Evolution, 28(3), 167–177. 10.1016/j.tree.2012.10.004 23141923

[ece35823-bib-0060] Oberdoff, T. , Guégan, J. F. , & Hugueny, B. (1995). Global scale patterns of fish species richness in rivers. Ecography, 18(4), 345–352. 10.1111/j.1600-0587.1995.tb00137.x

[ece35823-bib-0061] Pagotto, J. P. A. , Goulart, E. , Oliveira, E. F. , & Yamamura, C. B. (2011). Trophic ecomorphology of Siluriformes (Pisces, Osteichthyes) from a tropical stream. Brazilian Journal of Biology, 71(2), 469–479. 10.1590/S1519-69842011000300017 21755165

[ece35823-bib-0062] Pavoine, S. , & Bonsall, M. B. (2011). Measuring biodiversity to explain community assembly: A unified approach. Biological Reviews, 86(4), 792–812. 10.1111/j.1469-185X.2010.00171.x 21155964

[ece35823-bib-0063] Perronne, R. , Munoz, F. , Borgy, B. , Reboud, X. , & Gaba, S. (2017). How to design trait‐based analyses of community assembly mechanisms: Insights and guidelines from a literature review. Perspectives in Plant Ecology, Evolution and Systematics, 25, 29–44. 10.1016/j.ppees.2017.01.004

[ece35823-bib-0064] Pianka, E. R. , Vitt, L. J. , Pelegrin, N. , Fitzgerald, D. B. , & Winemiller, K. O. (2017). Toward a periodic table of niches, or exploring the lizard niche hypervolume. The American Naturalist, 19(5), 601–616. 10.1086/693781 29053363

[ece35823-bib-0065] Poff, N. L. (1997). Landscape filters and species traits: Towards mechanistic understanding and prediction in stream ecology. Journal of the North American Benthological Society, 16(2), 391–409. 10.2307/1468026

[ece35823-bib-0066] Poff, N. L. , & Allan, J. D. (1995). Functional organization of stream fish assemblages in relation to hydrological variability. Ecology, 76(2), 606–627. 10.2307/1941217

[ece35823-bib-0067] Rabosky, D. L. , Chang, J. , Title, P. O. , Cowman, P. F. , Sallan, L. , Friedman, M. , …, & Alfaro, M. E. (2018). An inverse latitudinal gradient in speciation rate for marine fishes. Nature, 559(7714), 392. 29973726 10.1038/s41586-018-0273-1

[ece35823-bib-0068] Ramm, T. , Cantalapiedra, J. L. , Wagner, P. , Penner, J. , Rödel, M. O. , & Müller, J. (2018). Divergent trends in functional and phylogenetic structure in reptile communities across Africa. Nature Communications, 9(1), 4697 10.1038/s41467-018-07107-y PMC6224532 30409973

[ece35823-bib-0069] Resh, V. H. , Brown, A. V. , Covich, A. P. , Gurtz, M. E. , Li, H. W. , Minshall, G. W. , … Wissmar, R. C. (1988). The role of disturbance in stream ecology. Journal of the North American Benthological Society, 7(4), 433–455. 10.2307/1467300

[ece35823-bib-0070] Ross, S. T. , & Brenneman, W. M. (2001). The inland fishes of Mississippi. Jackson, Mississippi: Univ. Press of Mississippi.

[ece35823-bib-0071] Schemske, D. W. , Mittelbach, G. G. , Cornell, H. V. , Sobel, J. M. , & Roy, K. (2009). Is there a latitudinal gradient in the importance of biotic interactions? Annual Review of Ecology, Evolution, and Systematics, 40, 245–269. 10.1146/annurev.ecolsys.39.110707.173430

[ece35823-bib-0072] Smith, A. B. , Sandel, B. , Kraft, N. J. , & Carey, S. (2013). Characterizing scale‐dependent community assembly using the functional‐diversity–area relationship. Ecology, 94(11), 2392–2402. 10.1890/12-2109.1 24400491

[ece35823-bib-0073] Swenson, N. G. (2013). The assembly of tropical tree communities–the advances and shortcomings of phylogenetic and functional trait analyses. Ecography, 36(3), 264–276. 10.1111/j.1600-0587.2012.00121.x

[ece35823-bib-0074] Swenson, N. G. , & Enquist, B. J. (2007). Ecological and evolutionary determinants of a key plant functional trait: Wood density and its community‐wide variation across latitude and elevation. American Journal of Botany, 94(3), 451–459. 10.3732/ajb.94.3.451 21636415

[ece35823-bib-0075] Swenson, N. G. , & Enquist, B. J. (2009). Opposing assembly mechanisms in a Neotropical dry forest: Implications for phylogenetic and functional community ecology. Ecology, 90(8), 2161–2170. 10.1890/08-1025.1 19739378

[ece35823-bib-0076] Swenson, N. G. , Enquist, B. J. , Pither, J. , Thompson, J. , & Zimmerman, J. K. (2006). The problem and promise of scale dependency in community phylogenetics. Ecology, 87(10), 2418–2424. 10.1890/0012-9658(2006)87[2418:TPAPOS]2.0.CO;2 17089650

[ece35823-bib-0077] Tokeshi, M. , & Arakaki, S. (2012). Habitat complexity in aquatic systems: Fractals and beyond. Hydrobiologia, 685(1), 27–47. 10.1007/s10750-011-0832-z

[ece35823-bib-0078] Trisos, C. H. , Petchey, O. L. , & Tobias, J. A. (2014). Unraveling the interplay of community assembly processes acting on multiple niche axes across spatial scales. The American Naturalist, 184(5), 593–608. 10.1086/678233 25325744

[ece35823-bib-0079] Troia, M. J. , & Gido, K. B. (2015). Functional strategies drive community assembly of stream fishes along environmental gradients and across spatial scales. Oecologia, 177(2), 545–559. 10.1007/s00442-014-3178-1 25502608

[ece35823-bib-0080] Vamosi, S. M. , Heard, S. B. , Vamosi, J. C. , & Webb, C. O. (2009). Emerging patterns in the comparative analysis of phylogenetic community structure. Molecular Ecology, 18(4), 572–592. 10.1111/j.1365-294X.2008.04001.x 19037898

[ece35823-bib-0081] Violle, C. , Navas, M. L. , Vile, D. , Kazakou, E. , Fortunel, C. , Hummel, I. , & Garnier, E. (2007). Let the concept of trait be functional!. Oikos, 116(5), 882–892. 10.1111/j.2007.0030-1299.15559.x

[ece35823-bib-0082] Violle, C. , Reich, P. B. , Pacala, S. W. , Enquist, B. J. , & Kattge, J. (2014). The emergence and promise of functional biogeography. Proceedings of the National Academy of Sciences of the United States of America, 111(38), 13690–13696. 10.1073/pnas.1415442111 25225414 PMC4183284

[ece35823-bib-0083] Webb, C. O. , Ackerly, D. D. , McPeek, M. A. , & Donoghue, M. J. (2002). Phylogenies and community ecology. Annual Review of Ecology and Systematics, 33(1), 475–505. 10.1146/annurev.ecolsys.33.010802.150448

[ece35823-bib-0084] Webb, P. W. (1984). Form and function in fish swimming. Scientific American, 251(1), 72–83. 10.1038/scientificamerican0784-72

[ece35823-bib-0085] Webb, P. W. (1988). ‘Steady'swimming kinematics of tiger musky, an esociform accelerator, and rainbow trout, a generalist cruiser. Journal of Experimental Biology, 138(1), 51–69.

[ece35823-bib-0086] Weiher, E. , Freund, D. , Bunton, T. , Stefanski, A. , Lee, T. , & Bentivenga, S. (2011). Advances, challenges and a developing synthesis of ecological community assembly theory. Philosophical Transactions of the Royal Society B: Biological Sciences, 366(1576), 2403–2413. 10.1098/rstb.2011.0056 PMC3130429 21768155

[ece35823-bib-0087] Weiher, E. , & Keddy, P. A. (1995). Assembly rules, null models, and trait dispersion: New questions from old patterns. Oikos, 159–164. 10.2307/3545686

[ece35823-bib-0088] Wiens, J. J. , Ackerly, D. D. , Allen, A. P. , Anacker, B. L. , Buckley, L. B. , Cornell, H. V. , … Stephens, P. R. (2010). Niche conservatism as an emerging principle in ecology and conservation biology. Ecology Letters, 13(10), 1310–1324. 10.1111/j.1461-0248.2010.01515.x 20649638

[ece35823-bib-0089] Wiens, J. J. , & Graham, C. H. (2005). Niche conservatism: Integrating evolution, ecology, and conservation biology. Annual Review of Ecology, Evolution, and Systematics, 36, 519–539. 10.1146/annurev.ecolsys.36.102803.095431

[ece35823-bib-0090] Willis, S. C. , Winemiller, K. O. , & Lopez‐Fernandez, H. (2005). Habitat structural complexity and morphological diversity of fish assemblages in a Neotropical floodplain river. Oecologia, 142(2), 284–295. 10.1007/s00442-004-1723-z 15655689

[ece35823-bib-0091] Winemiller, K. O. (1991). Ecomorphological diversification in lowland freshwater fish assemblages from five biotic regions. Ecological Monographs, 61(4), 343–365. 10.2307/2937046

[ece35823-bib-0092] Winemiller, K. O. , Fitzgerald, D. B. , Bower, L. M. , & Pianka, E. R. (2015). Functional traits, convergent evolution, and periodic tables of niches. Ecology Letters, 18(8), 737–751. 10.1111/ele.12462 26096695 PMC4744997

