# Supplementary figures and images for: Intercontinental trends in functional and phylogenetic structure of stream fish assemblages
Source: Ecol Evol. 2019 Nov 19;9(24):13862–76. doi: 10.1002/ece3.5823 (PMC6953669; doi:10.1002/ece3.5823)

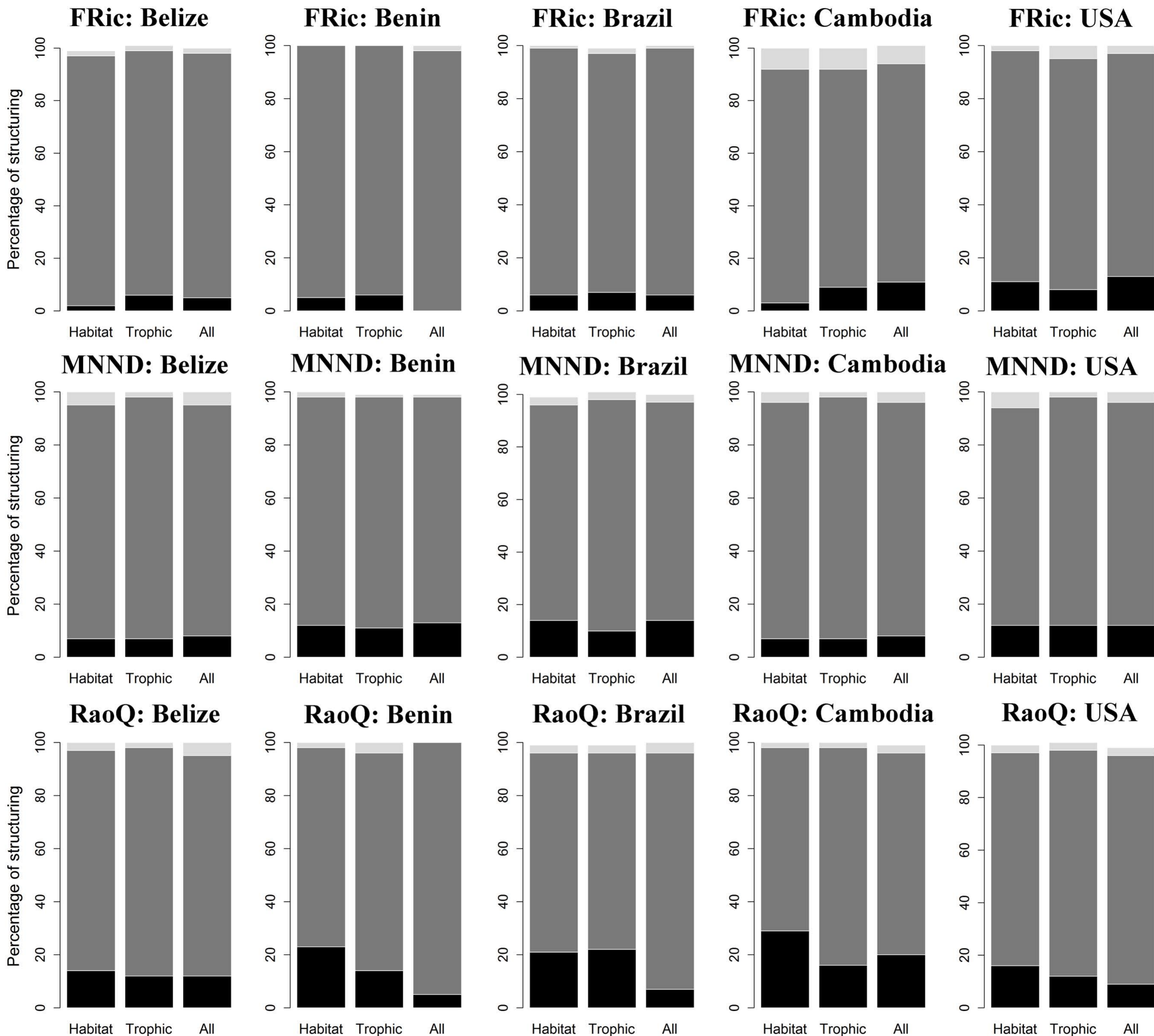

Supplement: Supplementary file 1 [file ECE3-9-13862-s001.pdf]

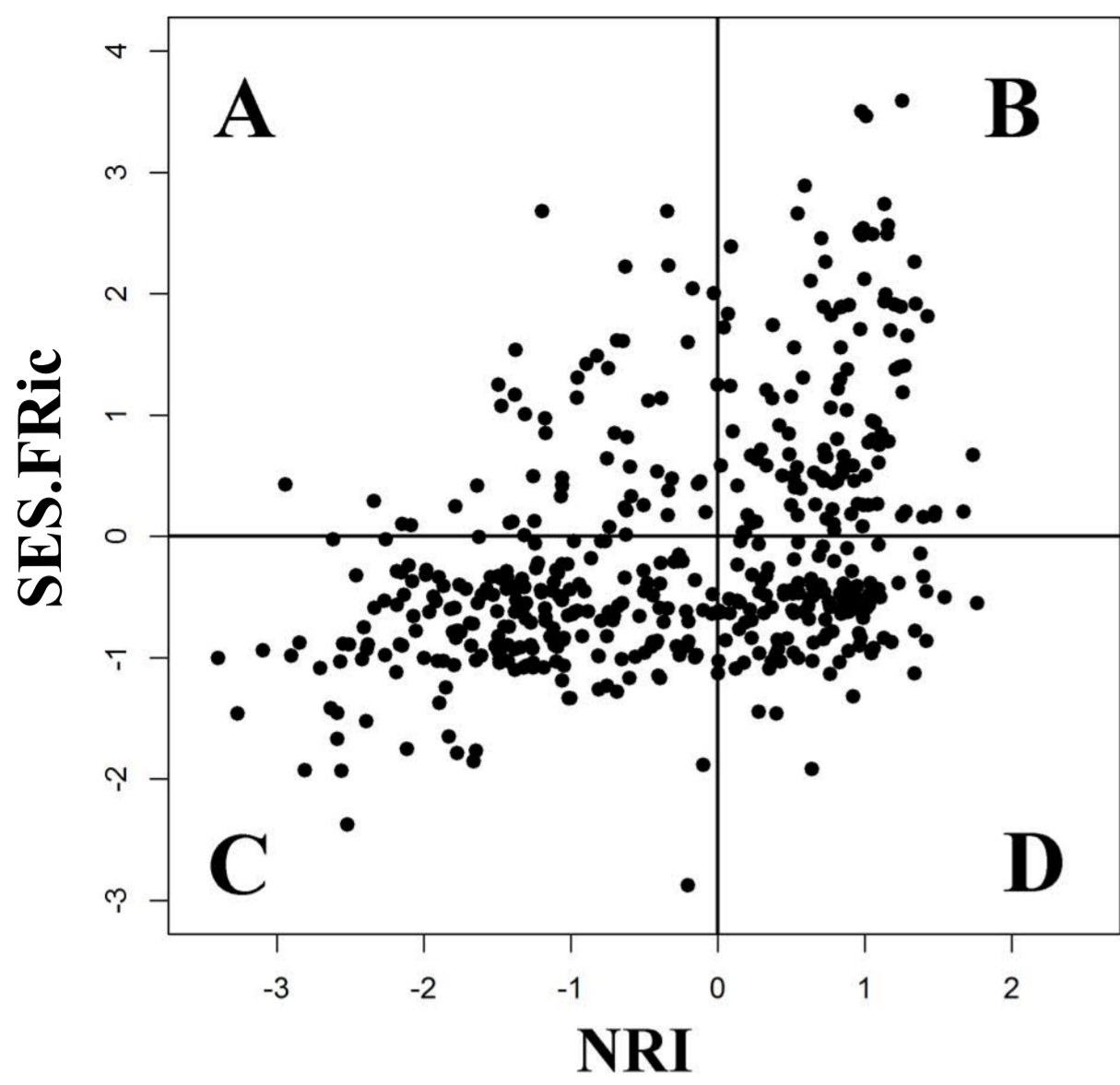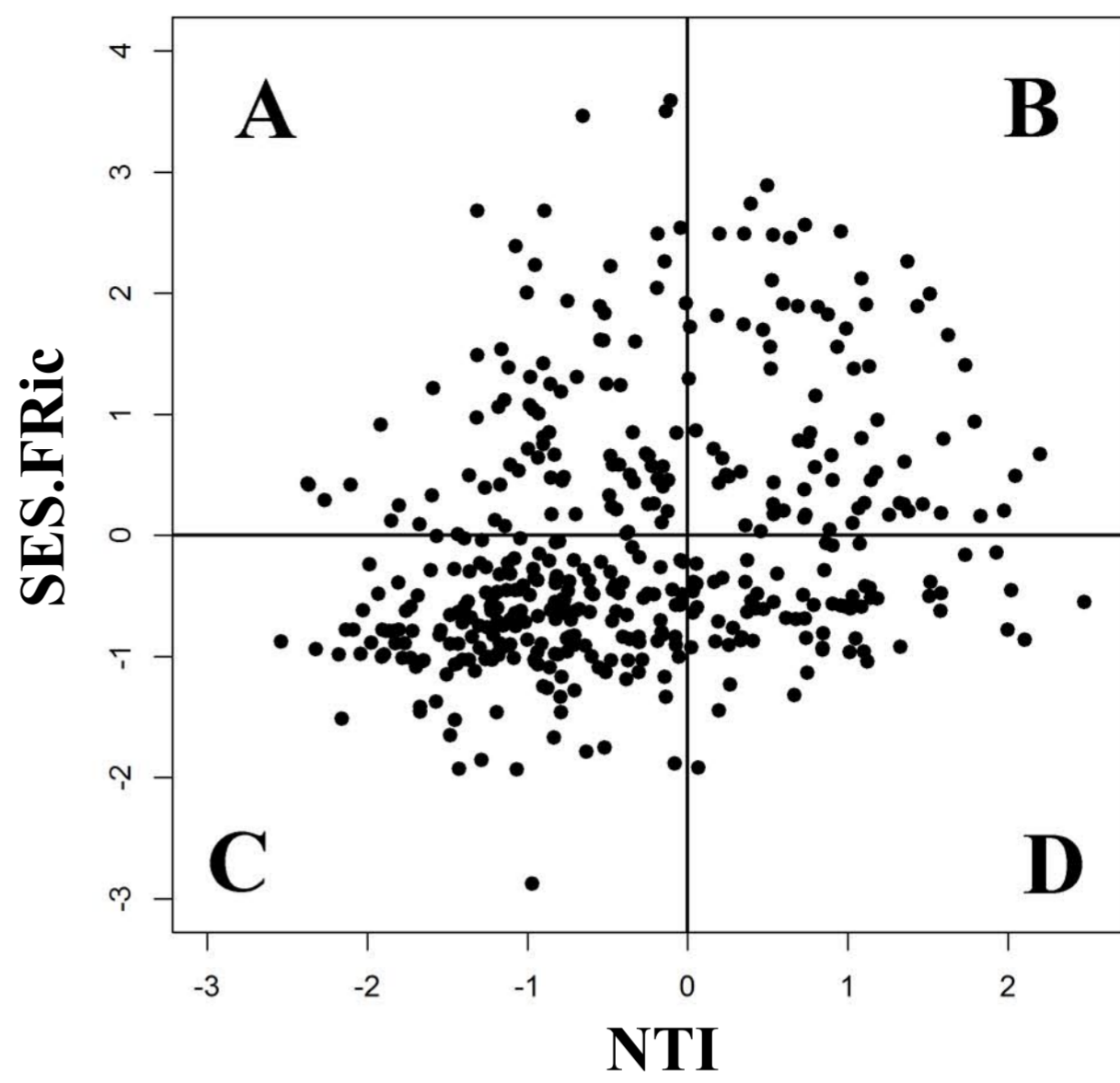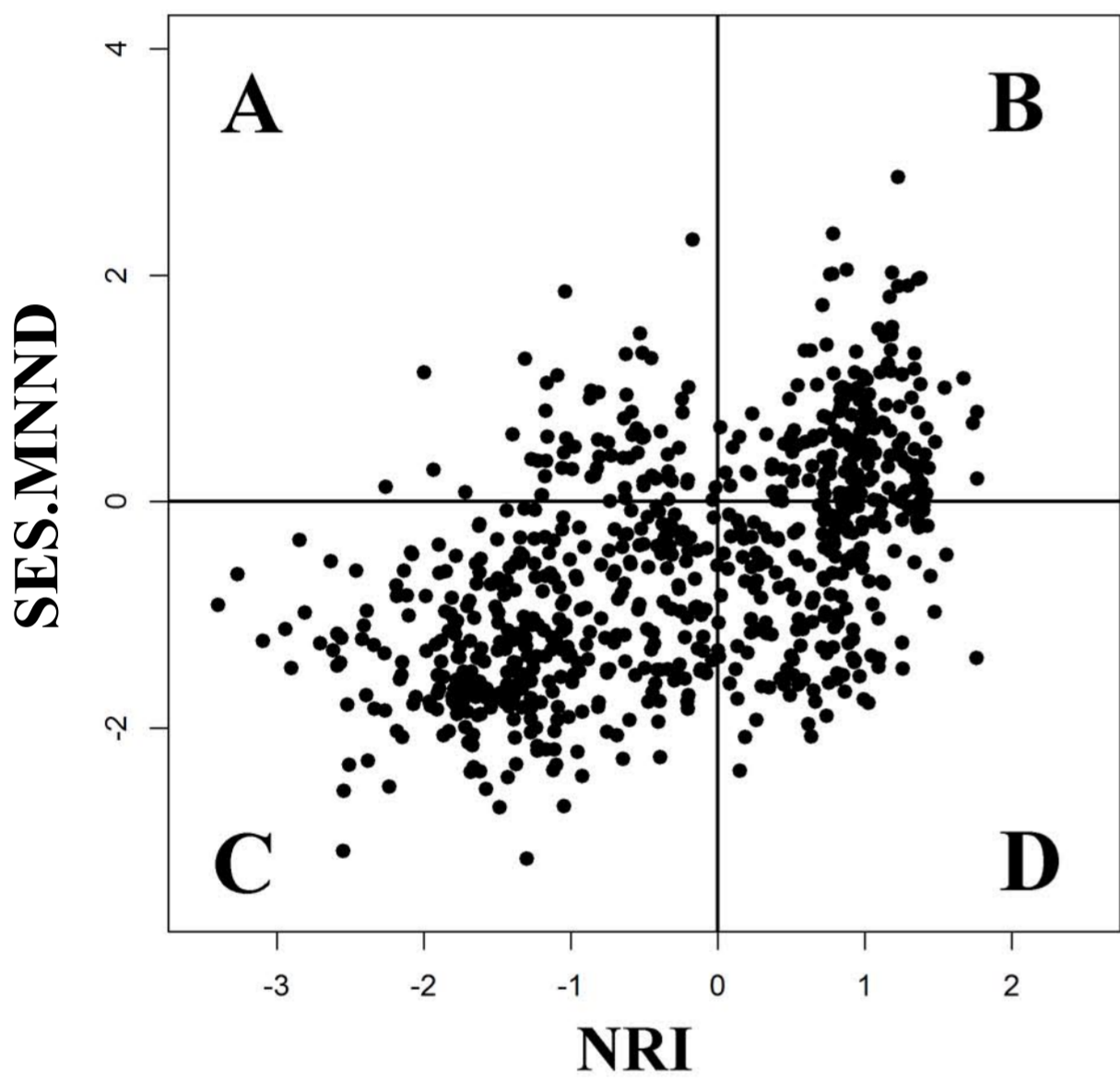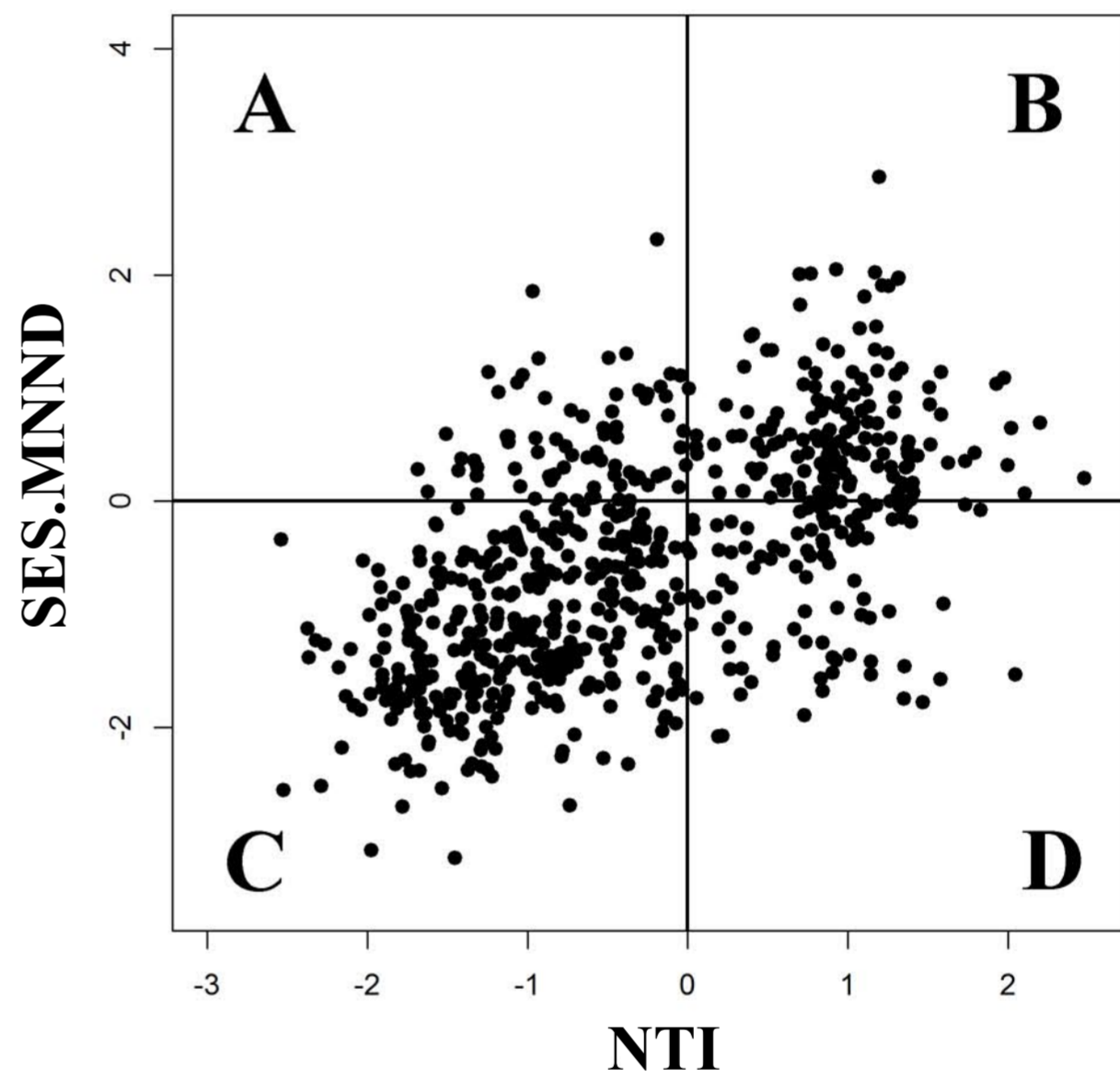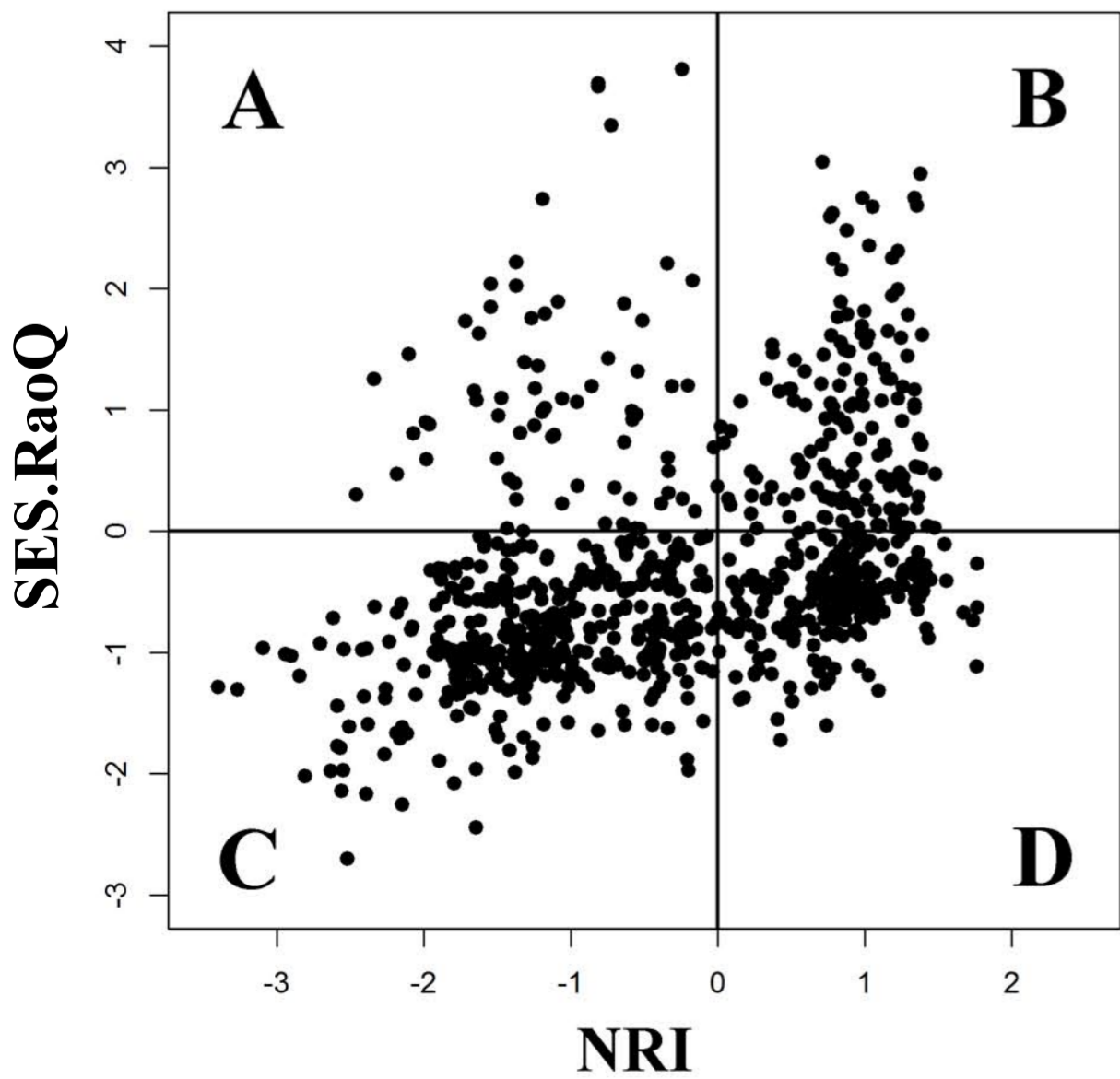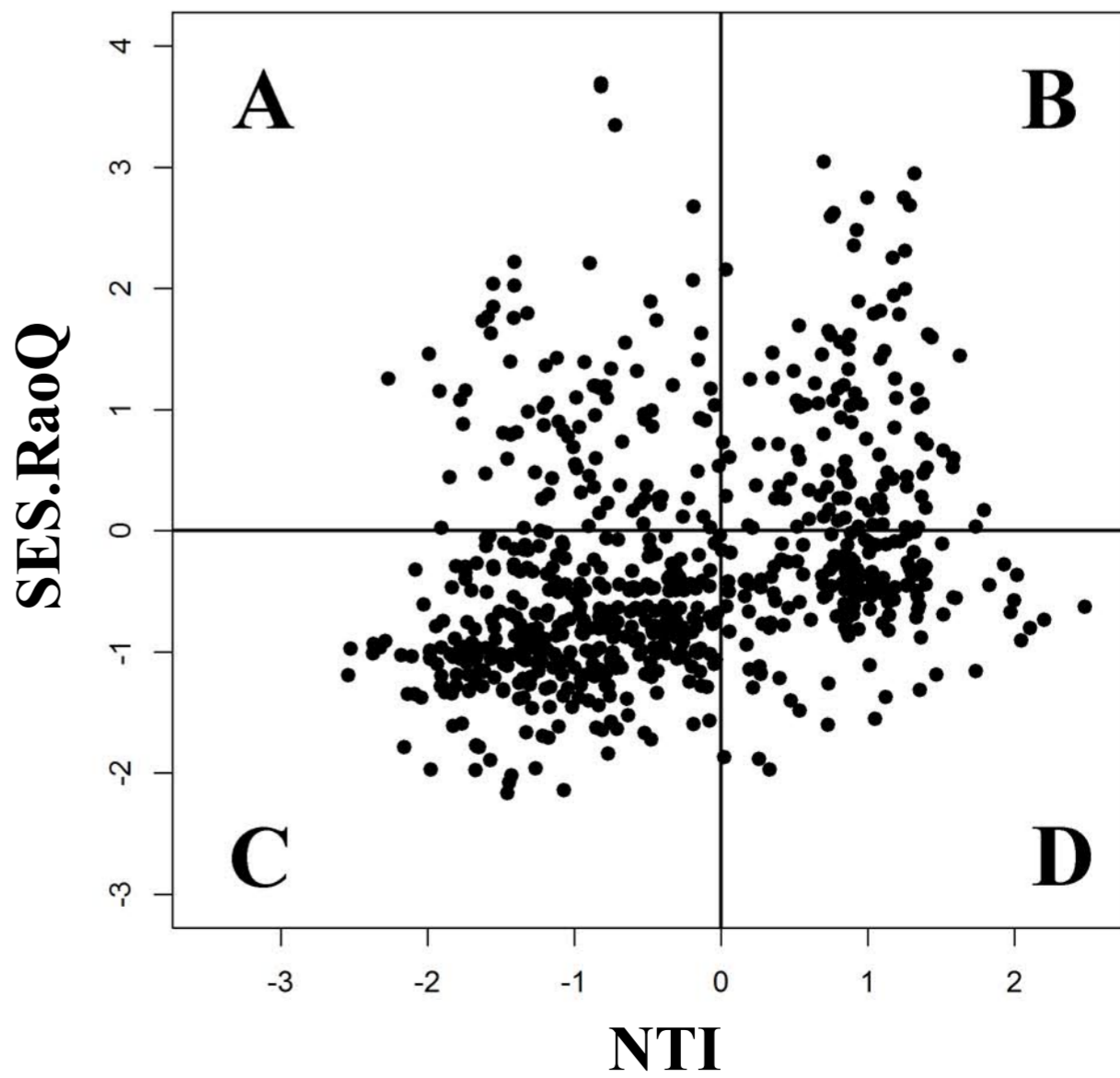

Supplement: Supplementary file 2 [file ECE3-9-13862-s002.pdf]

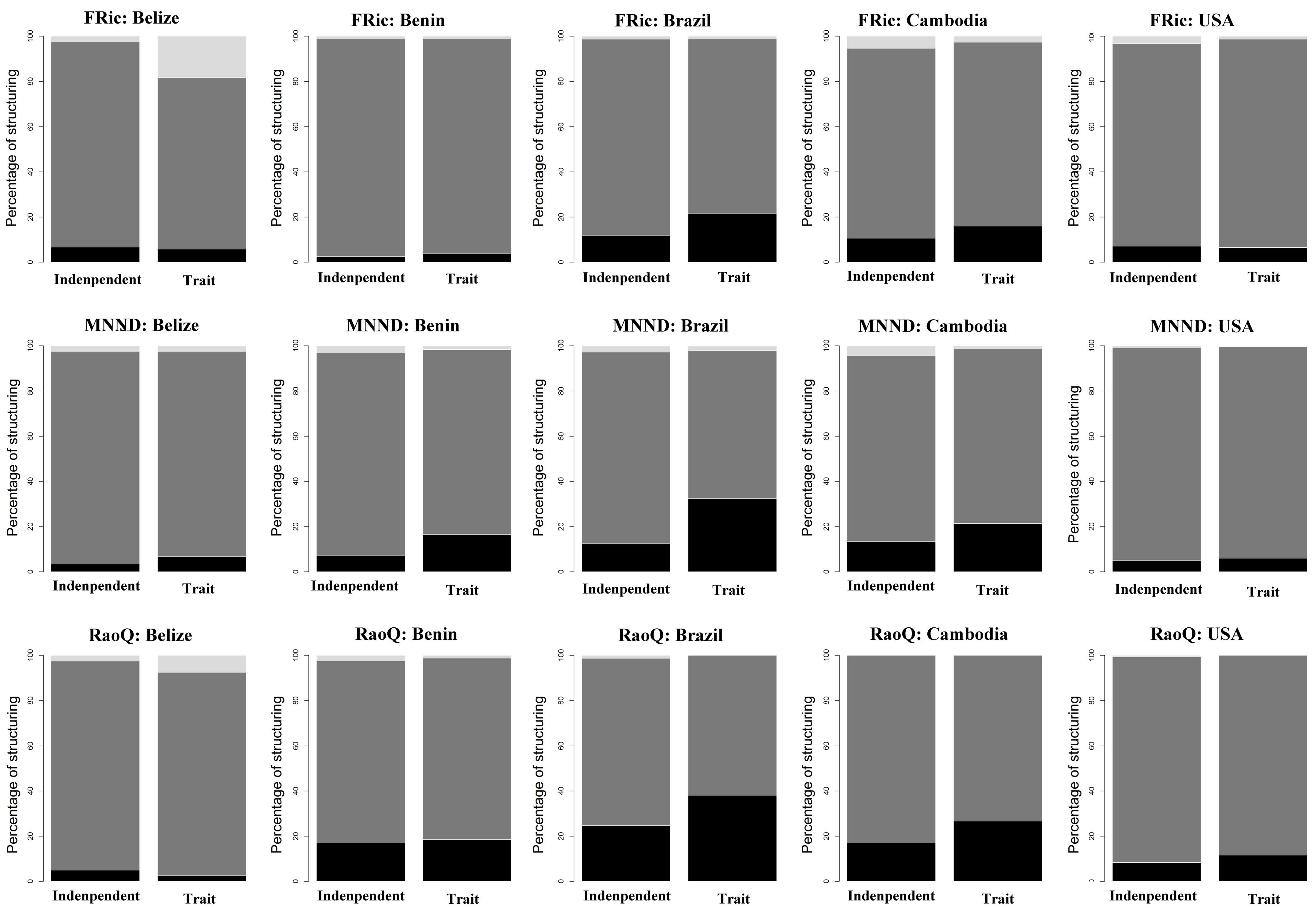

Supplement: Supplementary file 3 [file ECE3-9-13862-s003.pdf]
