# Supplementary material for: Intercontinental trends in functional and phylogenetic structure of stream fish assemblages
Source: Ecol Evol. 2019 Nov 19;9(24):13862–76. doi: 10.1002/ece3.5823 (PMC6953669; doi:10.1002/ece3.5823)
Supplement: Supplementary file 9 [file ECE3-9-13862-s009.docx]

Figure S1 Functional trait diversity for each region based on independent-swap model and all three metrics: FRic, MNND, and RaoQ. Proportions of significantly overdispersed (light gray), underdispersed (black), and randomly (dark gray) structured local assemblages at the microhabitat scale using the corresponding stream reach as the regional species pool

Figure S2 Standardized effect size (SES) for FRic, MNND, or RaoQ plotted against NTI or NRI based on the taxon-label model using diet traits. Lines divide plot into quadrats. Quadrat (a) suggests morphological divergence and niche segregation of related species; (b) morphological divergence and niche segregation of unrelated species; (c) morphological underdispersion of related species due to stabilizing selection or niche conservatism; (d) morphological convergence of unrelated species reflecting habitat filtering of convergent forms

Figure S3 Functional trait diversity based on feeding traits for each region based on independent-swap (Independent) and taxon-label (Labels) models and all three metrics: FRic, MNND, and RaoQ. Proportions of significantly overdispersed (light gray), underdispersed (black), and randomly (dark gray) structured local assemblages at the microhabitat scale using the corresponding stream reach as the regional species pool
